# Synthesis, Structure, DNA/BSA Binding, DNA Cleaving, Cytotoxic and SOD Mimetic Activities of Copper(II) Complexes Derived from Methoxybenzylamine Schiff Base Ligands

**DOI:** 10.3390/molecules30173461

**Published:** 2025-08-22

**Authors:** Lucia Lintnerová, Peter Herich, Jana Korcová, Barbora Svitková, Flóra Jozefíková, Jindra Valentová

**Affiliations:** 1 Department of Chemical Theory of Drugs, Faculty of Pharmacy, Comenius University in Bratislava, 832 32 Bratislava, Slovakia; peter.herich@stuba.sk (P.H.); flora.jozefikova@stuba.sk (F.J.); 2 Institute of Physical Chemistry, Faculty of Chemical and Food Technology, Slovak University of Technology, 812 37 Bratislava, Slovakia; 3 Cancer Research Institute, Biomedical Research Center, Slovak Academy of Sciences, 845 05 Bratislava, Slovakia; barbora.svitkova@savba.sk; 4 Institute of Inorganic Chemistry, Technology and Materials, Faculty of Chemical and Food Technology, Slovak University of Technology, 812 37 Bratislava, Slovakia

**Keywords:** copper(II) complexes, DNA binding, BSA binding, DNA cleavage, cytotoxicity, SOD mimetics, Schiff bases

## Abstract

Schiff base ligands prepared from salicylaldehyde and 2-, 3- and 4-methoxybenzylamine were used to prepare copper(II) complexes, characterized by spectral methods, elemental analysis and X-ray crystallography in the case of complex **4a** derived from 2-methoxybenzylamine. The DNA cleavage activity of the prepared complexes was exceptional, with best activities of over 95% one-strand cleavage for **4c** at 3 mM and full double-strand cleavage for complex **4a** at 5 mM. Absorption titration studies with ct-DNA revealed good binding constants (at 10^5^ M^−1^) with a decrease of up to 56% light absorption. Meanwhile, the EB–DNA displacement method and viscosity studies revealed groove binding as a possible binding mode. For BSA binding studies, all three complexes showed K_BSA_ values in the optimal range for reversible BSA binding (10^4^ M^−1^). The copper(II) complexes showed significant cytotoxic effects (67–96% at 1 mM) in mitochondrial activity monitoring assays. Cytotoxicity was confirmed against cancer cell lines (A549 and HepG2) and HEL cells. The complexes **4a** and **4c** exhibited high activity against HepG2 cancer cells (IC50 < 22 μM), comparable to cisplatin. The radical scavenging activity was determined by the INT method with the best IC50 for **4c** (189 ± 11 μM). Overall, complexes **4a** and **4c** with a methoxy group in the *ortho* and *para* positions show high potential in most determined activities, but mainly as DNA cleavers and as cytotoxic agents with selectivity against HepG2 cells.

## 1. Introduction

The binding ability of metal complexes to DNA is crucial for the development of new metal-based chemotherapeutics and/or cytotoxic agents. Binding on its own can affect the proliferation of cells; this has been shown with platinum-based anticancer drugs. Alternatives based on copper (but also other transition metals such as nickel, manganese, zinc, iron and others) have been studied excessively because of their lower toxicity, and biocompatibility, but also their broader variety of activities due to their important role in many biological systems such as metalloenzymes. Specifically, copper possesses altered metabolism in cancer cells compared to normal cells, and it can also be found in higher amounts in cancer cells. The cancer cell might therefore be a selective target for copper-based drugs [1,2,3]. The mechanism of action of copper in cancer cells is not fully clear, but studies from over the past several years have suggested several pathways, such as angiogenesis, inhibition of proteasome activity, modulation of ROS levels in cells or apoptosis induction targeting caspases or DNA damage [4].

The anticancer mechanism of action of copper(II) complexes includes binding to DNA mostly with noncovalent interactions (various types of intercalations, major or minor groove binding or electrostatic interactions with the phosphate/sugar backbone of DNA) compared to cisplatin and its derivatives binding via covalent bonds. Additionally, the binding is also linked to oxidative cleavage induced by reactive oxygen species (ROS) generation or hydrolytic cleavage of DNA. Due to their DNA binding and cleaving properties, copper complexes are suitable antitumor agents but can also serve as DNA markers and promoters and DNA foot-printing agents [5,6,7,8].

Structural properties such as the ligand shape, size and structure and coordination geometry are key factors for the strength of DNA binding. The planar shape of ligands is especially important since it allows deeper insertion into the DNA double helix for stronger binding. The binding of the complex to DNA leads in many cases to deformation of the DNA structure, making cleavage possible, but also to DNA sequence modification. The result may be inhibition of protein synthesis, mitochondrial activity [9] or cell proliferation. Copper-based antineoplastic drugs function as chemical nucleases that can oxidize nucleic bases or deoxyribose rings and hydrolyze phosphate esters [10].

Schiff bases derived from aromatic aldehydes with an electron-rich *ortho* substitution such as salicylaldehyde and its derivatives, *N*-substituted 2-aminobenzaldehydes or 3-hydroxy-naphthalene-2-carbaldehyde and its derivatives, etc., also provide suitable chelating binding for copper(II) and other metals. They can be modified easily by choosing different aldehyde/amine partners in the condensation reaction and metal center allowing fine-tuning of the biological properties of the ligands and complexes [3,11,12].

Schiff base metal complexes have been the subject of an enormous number of studies. Due to the relatively simple synthesis and metal-binding capabilities of Schiff bases, their complexes have garnered significant attention for their unique structural, biological and chemical properties. The conjugation of the C=N bond with aromatic systems or other double bonds allows for preparation of complexes with more predictable, partially planar coordination geometry, which is useful in drug design. The biological activities of Schiff base copper(II) complexes range from antibacterial, antifungal, antiradical, anticancer, antioxidant, antidiabetic properties and enzyme inhibition [12,13,14,15]. Their anticancer activities are mainly based on DNA binding and cleaving properties [6,11,16,17].

Copper complexes can undergo a fast oxidation state change between +II and +I and back by reacting with free radicals as superoxide dismutase (SOD) mimetics but they also can generate radicals (ROS) by releasing or accepting one electron. Both effects can be used in cancer therapy, either as a prevention of radical formation or as cytotoxic agents within cancer cells capable of gathering greater amounts of copper within them [18,19,20,21].

SOD mimetics are metal complexes able to turn superoxide (O_2_^−^) by oxidation to oxygen (O_2_) and by reduction to the peroxide anion (O_2_^−2^) while the redox active metal center such as Cu or Mn undergoes a change in oxidative state (II/I or III/II). The main application of SOD mimetics is their ability to absorb or release one electron, with respect to scavenge radicals or reactive oxygen species (ROS) [22,23,24,25]. ROS form naturally in the organism, mainly in mitochondria during metabolism, and their main role is in immunity and defense processes against pathogens. Their overproduction as a result of external sources, infections, increased production of uric acid, autooxidation of thiols, hyperglycemia, etc., can lead to pathological states and the development of major diseases such as cancer, atherosclerosis, stroke, asthma, heart attacks, retina damage, diabetes mellitus, etc. [26,27].

In this study, a series of Schiff base ligands derived from 2-, 3- and 4-methoxybenzylamines and salicylaldehyde were prepared and used in the synthesis of stereoisomeric ligand copper(II) complexes. This study is dedicated to the regio-isomerism of the methoxy group on the benzylamine moiety of the ligand molecule and its effect on the anticancer activities of the complexes. For this purpose, DNA binding and DNA cleaving assays, resazurin cell viability and tumor cell cytotoxicity MTT assays were performed. A bovine serum albumin (BSA) binding assay of the prepared complexes was performed to determine potential drug delivery to a solid tumor; alternatively, their SOD mimetic activities were studied using the INT radical scavenging method.

## 2. Results and Discussion

### 2.1. Synthesis

**Scheme 1:**
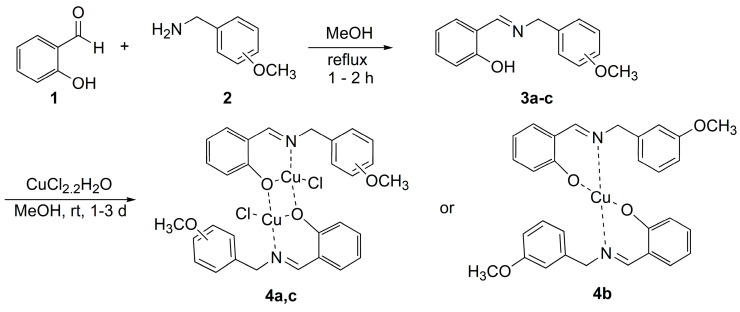
Overall synthesis of Schiff bases **3a-c** and their copper(II) complexes **4a**–**c** (**a**: 2-methoxy-, **b**: 3-methoxy-, **c**: 4-methoxyderivative).

The synthesis of Schiff bases **3a**–**c** started from salicylaldehyde (**1**) and 2-,3- and 4-methoxybenzylamine (**2a**–**c**). The procedures were performed analogously. The reaction mixture was refluxed in dry methanol for 1–2 h (Scheme 1). The progress of the reaction could be observed by a change in color to yellow and TLC analysis. Schiff base products were isolated by evaporation and washed with diethyl ether as yellow oils and kept under anhydrous conditions.

The complexes of Schiff bases **3a**–**c** were prepared in methanol using copper(II) chloride, a copper(II) compound soluble in methanol without the need for heating. We wanted to avoid heating in this step to reduce any chance of decomposition of **3a**–**c**. Complexes **4a**–**c** were isolated as dark green powders (**4b** and **4c**) or fine dark green crystals (**4a**). Complex **4b** had a less usual ligand/copper ratio (2:1), which was discovered by elemental analysis and confirmed by MS studies (Supplementary Data); the other two had the expected 2 : 2 ratios confirmed by X-ray analysis for **4a**. We expected **4c** to have an analogous structure.

### 2.2. X-Ray Structure of Complex ***4a***

**Figure 1:**
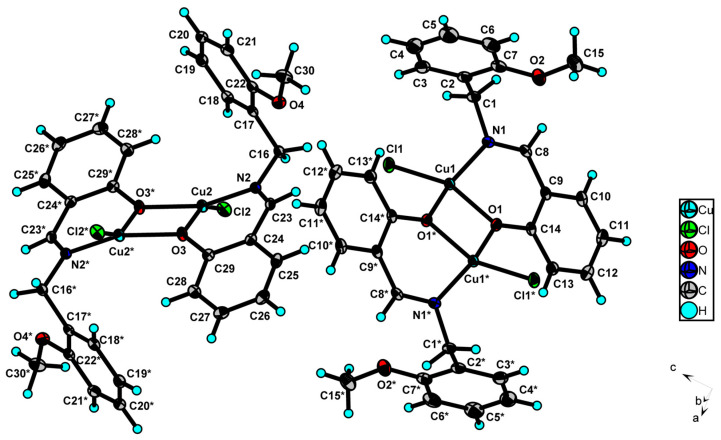
The molecular structure (as two dimer molecules) of complex **4a** showing the atom-labeling scheme without hydrogen atom labels for clarity. Displacement ellipsoids are drawn at the 50% probability level.

Complex **4a** crystallized in the monoclinic P2_1_/c space groups (No. 14) as two independent molecules (Figure 1 and Figure S1-1). In the cell unit, there are 14 molecules (Figure S1-2). Complex **4a** consists of two Schiff base ligands and chloro ligands bonded over central atoms. The complex is created from dimeric molecules with disordered square-planar arrangement around the central atoms. Each copper center atom (Cu1/Cu1*) binds by a six-membered chelate ring to one Schiff base ligand (O1,N1) and also has a bond with phenolic oxygen O1* on the second Schiff base molecule. This coordination mode forms the four-membered ring at the center. The fourth coordination bond is with the chloro ligand. The distance of the central Cu1 atom from the plane given by the O1*O1N1 atoms is 0.409(2) Å and distance of Cl1 atom from same plan is 1.818(3) Å. The second molecule has a similar distance of the central Cu2 atom from the plane given by the O3*O3N2 atoms; this distance is 0.422(2) Å and the distance of the Cl2 atom from the same plane is 1.879(3) Å. The crystallographic data for complex **4a** are summarized in Table 1. Selected bond lengths and angles are listed in Table 2.

The stacking of molecules in crystal structure of complex **4a** is caused by a system of intermolecular hydrogen bonds and interactions (Figure S1-3, Table S1-1).

### 2.3. EPR Studies

**Figure 2:**
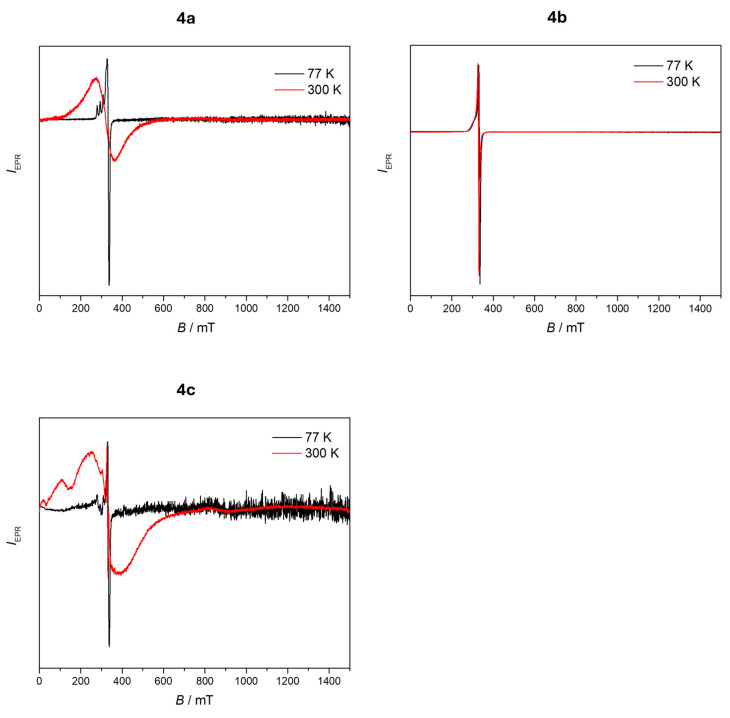
Solid-state powder EPR spectra of **4a**–**c** at 300 K (red line) and at 77 K (black line).

At 300 K, the EPR spectrum of dinuclear complex **4a** shows a very broad complex signal as the result of exchange/dipole interaction between unpaired copper electrons (Figure 2). Small, almost invisible teeth at approximately 300 mT are probably an axial spectrum component as the result of non-perfect spin–spin coupling (spin defect). This could be the result of the copper–copper distance or non-optimal geometry. At 77 K, a standard axial copper spectrum with four-line parallel component hyperfine splitting from the copper nucleus is observed, and the broad signal dominating at higher temperatures disappeared at 77 K. This suggests antiferromagnetic coupling between two copper atoms, an exchange-broadened spectrum at room temperature and the formation of an EPR silent singlet state at low temperatures. The observation of axial EPR spectra at 77 K, as well as small teeth in the spectrum at 300 K, confirms non-perfect spin–spin coupling.

As expected, derivative **4b** with only one copper atom in the molecule possesses a standard axial copper spectrum signal with unresolved hyperfine structure at both at room temperature and 77 K. There is no indication of any spin–spin interaction as proof of a mononuclear complex.

At 300 K, the EPR spectrum of complex **4c** shows a very broad complex signal with multiple signals. This is the result of the exchange reaction between unpaired copper electrons and ZFS (zero field splitting). A sharp signal at about 300 mT is probably an axial spectrum component and is more visible than that observed for the **4a** sample. Probably the interaction between copper atoms is slightly weaker than in the case of **4a**. At 77 K, a standard axial copper spectrum with four-line parallel component hyperfine splitting from the copper nucleus is observed, and the broad signal dominating at higher temperatures disappeared at 77 K. This suggests antiferromagnetic coupling between two copper atoms, as expected, similar to **4a**. Based on the EPR results, we can conclude that complex **4c** contains two paramagnetic copper atoms that can exist in an antiferromagnetic coupling.

### 2.4. DNA Interaction Studies

Many Schiff base copper complexes exert their anticancer activity by binding with DNA, thereby altering DNA replication and inhibiting the growth of tumor cells [10,28,29]. The diversity of the structure of metal complexes, different geometries, coordination numbers and redox potential influence the interaction with DNA, using various binding modes [30].

Copper complexes can interact with DNA via covalent and noncovalent interactions. The covalent binding mode involves the coordination of Cu^+2^ ions with nitrogen (N7) donor atoms of guanine and adenine or phosphate groups in the DNA backbone [10].

The predominately noncovalent binding mode between copper complexes and DNA includes π-π stacking interactions during intercalation, van der Waals interactions or hydrogen bonding associated with groove binding and electrostatic interactions [5]. Interactions of the prepared complexes **4a**–**c** with calf thymus DNA were evaluated using UV–Vis absorption titrations and fluorescence emission with the ethidium bromide (EB) displacement method.

#### 2.4.1. DNA Bnding Study by Absorption Titration

UV–Vis spectroscopic titration is routinely used to study the interaction of metal complexes with DNA and to distinguish possible binding modes. The DNA–complex interaction can be studied by comparison of UV–Vis absorption spectra of the free complex and the DNA–complex, which are usually different. Binding of the compound with DNA through intercalation usually results in hypochromism. In the case of electrostatic attraction between a compound and DNA, a hyperchromic effect appears [31].

**Figure 3:**
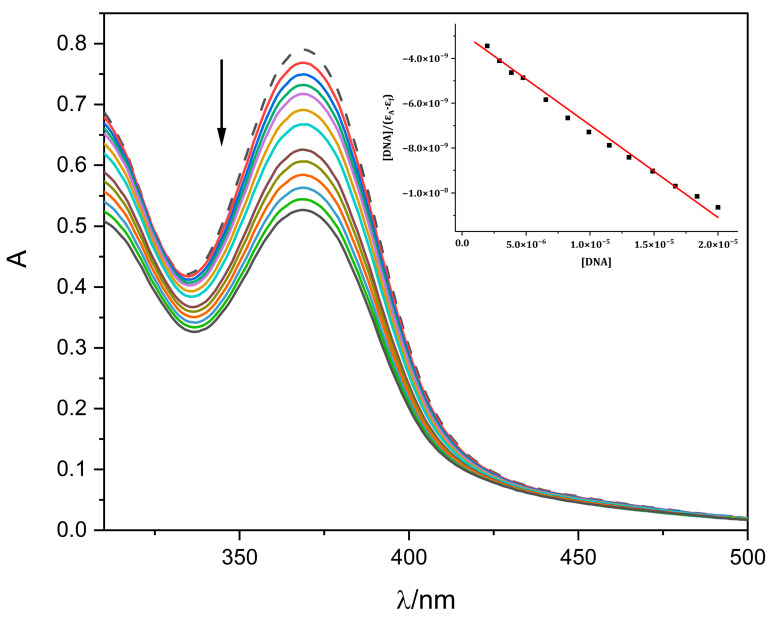
UV–Vis spectra of DMSO solution of complex **4a** in the absence (dashed line) and presence (solid line) of increasing amounts of ct-DNA (*r* = [DNA]/[complex] = 0–2.1). Arrows show changes in intensity upon the addition of increasing amounts of ct-DNA. Insert: Plot of [DNA]/ε_A_-ε_f_ vs. [DNA] for complex.

The absorption spectra of complexes **4a**–**c** (1 × 10^−4^ M^−1^) were recorded in the presence of increasing amounts of ct-DNA. Intraligand absorption bands with λ_max_ in the region 350 –380 nm were used to monitor the interaction of compounds with ct-DNA. The intraligand absorption value was chosen as λ_max_ = 258 nm, so the absorbance of DNA would not interfere. The UV–Vis spectrum affected by DNA addition is shown for complexes **4a** in Figure 3, and the UV–Vis spectra of other compounds are provided in the Supplementary Materials (Figure S3-1). In the UV spectra of **4a**–**c**, the intraligand absorption band of complexes (λ_max_ = 370 nm) exhibited hypochromism of 32, 26 and 28%, respectively. However, the gradual addition of ct-DNA to the solution of complexes did not cause a considerable shift in the absorption wavelengths. The spectral absorption characteristics of the studied complexes are shown in Table 3.

The DNA binding constants (K*_b_*) of the complexes were determined by the Wolfe–Shimer equation (Equation (1)) and the respective plots (Figure 3 and Figure S3-1). The K*_b_* constants are of the order of 10^5^, suggesting strong binding of the complexes to DNA. All three complexes showed significant interaction with DNA (K*_b_* = 1.4–3.2 × 10^5^).

**Figure 4:**
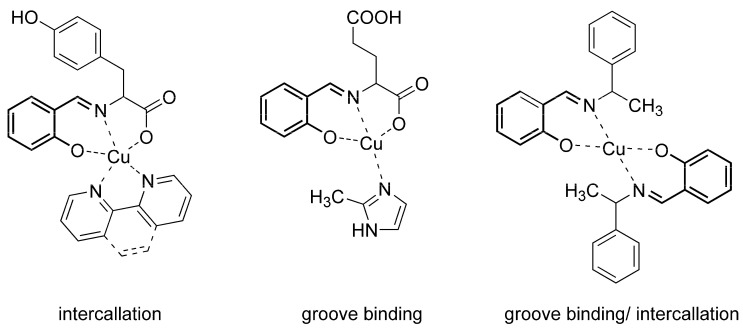
Closely related published DNA binders and their binding modes [32,33,34].

The binding constant values of **4a**–**c** are comparable to copper(II) Schiff base complexes derived from salicylaldehyde and various alpha amino acids with K_b_ in the 10^−3^ to 10^−4^ M^−1^ range [32,33] or other benzylamine derivatives with K_b_ in the 10^−5^ to 10^−6^ M^−1^ range [34]. This reflects the similar structure and planarity of the ligands (Figure 4). The conjugation effect of the imine double bond with the aromatic ring in Schiff bases and additional aromatic features in the ligand molecules creates a planar structural moiety that is beneficial for binding to DNA as intercalators or groove binders. The planarity of ligands could support the intercalation or partial intercalation binding mode of the tested complexes; the K*_b_* constant of complexes **4a**–**c** is close to that of the classical intercalator EB (K*_b_* = 1.23 × 10^5−^) [35]. It seems that the position of the substituent on the benzene ring in the ligand can influence the interaction with DNA. Based on the binding strength to DNA, the prepared complexes can be arranged as **4c** > **4a** ≈ **4b**, and the strongest effect is connected to the complex with the *para*-substituted benzene ring.

In addition to the possible intercalative mode, the metal complexes could bind to the minor groove of DNA by van der Waals interaction and hydrogen bonding [36]. However, the exact binding mode of complexes into DNA cannot be determined only by absorption titration studies, so further measurements are necessary to confirm the results obtained [31].

#### 2.4.2. Competitive Studies with EB–DNA

Competitive-binding experiments using the ethidium bromide (EB) displacement method from DNA may provide another confirmation of the binding affinity of the complexes. EB is a well-known DNA intercalator able to form an EB–DNA adduct by its insertion between adjacent DNA base pairs. The EB–DNA adduct is distinguished by an intense fluorescence emission band at 615 nm after excitation at 540 nm. Introducing a compound with DNA affinity leads to emission intensity decrease of the EB–DNA adduct as a result of the competition between the measured compound and EB for the binding sites within DNA.

**Figure 5:**
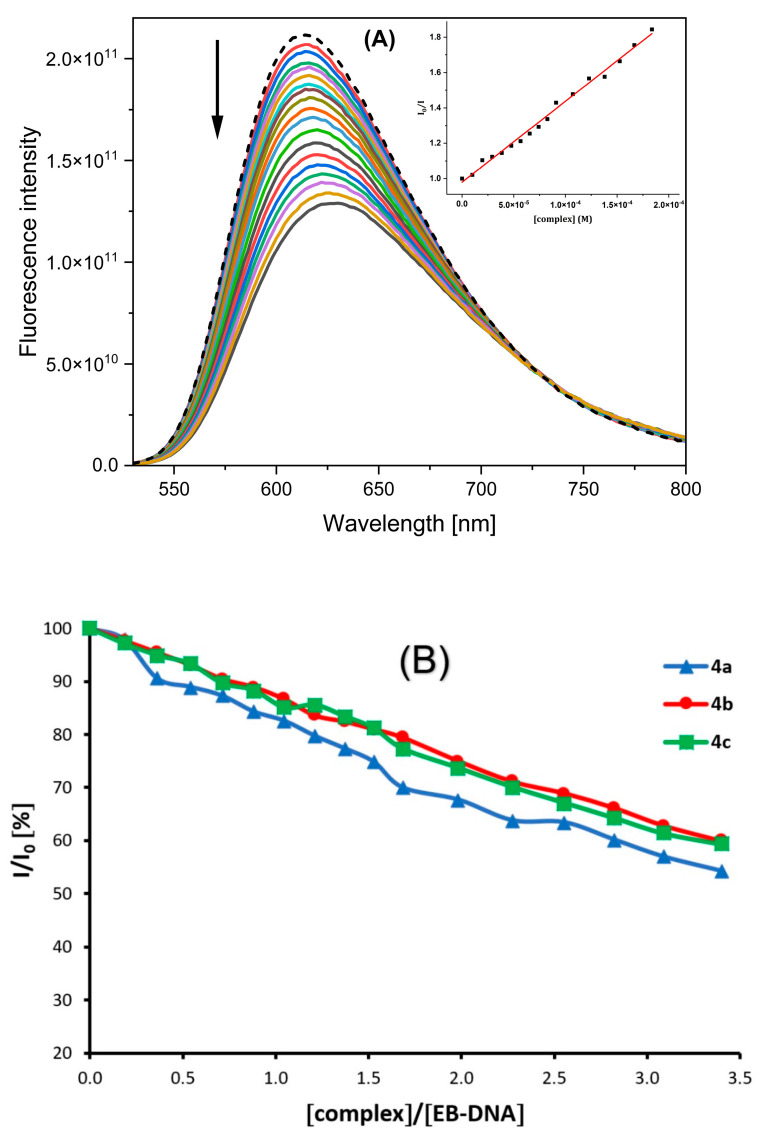
(**A**) Emission fluorescence spectra of EB–DNA in buffer solution in the presence of an increasing amount of complex **4a**. The concentration of the added complexes ranged from 0 to 0.16 mM (concentration increase 1.6 × 10^−2^ mM, indicated by a different color of the curve). The arrows indicate changes in fluorescence at increasing amounts of the complex. The insert graph shows the plot of I/I_0_ vs [complex]. (**B**) Plot of EB–DNA relative fluorescence intensity (I/I_0_), % at λ = 614 nm versus concentration ratio [complex]/[EB–DNA] for **4a**–**c**.

The fluorescence emission spectra of our studied complexes **4a**–**c** are shown in Figure 5A and Figure S3-2. The subsequent concentration increase of complexes led to a moderate decrease in the emission intensity band of the EB–DNA adduct. The maximum fluorescence quenching reached 40–45% of the initial EB–DNA product (Figure 5B, Table 4).

The quenching of EB–DNA fluorescence is in good agreement with the linear Stern–Volmer equation (Equations (2)–(4)) and the corresponding Stern–Volmer plots (Figure 5A and Figure S3-2) illustrate evidence of the interaction of tested complexes with DNA. The calculated *K_sv_* constant (3.61–4.58 × 10^3^) confirmed the moderate activity of complexes for EB–DNA fluorescence quenching and is comparable to other copper complexes with Schiff base ligands derived from salicylaldehyde and various amino acids [32,37].

According to the values of *K_sv_* (Table 4), the EB displacement ability of the studied Schiff base complexes was mutually comparable; however, the best effect was achieved in the complex containing the methoxy substituent in the *ortho* position (**4a**). Furthermore, the calculated values of the quenching rate constant *k*_q_ were higher than the value of 10^10^ M^−1^ s^−1^, indicating a static quenching mechanism that leads to the formation of a new adduct, i.e., between DNA and the complex [38].

Comparing the results obtained by both DNA binding methods reveals no clear common features among the prepared complexes, except for **4b** showing the lowest K*_b_* and K*_sv_*. The somewhat similar activity in EB–DNA displacement of complexes **4a**–**c** suggests a similar mode of action (intercalation, DNA groove binding or both). The competition between EB and the complexes suggests intercalation is low, based on the weak emission reduction [10]. This means that EB displacement occurs only partially, which is similar to some non-intercalating groove binders [39].

#### 2.4.3. Viscosity Measurements

To further verify the interaction mode of metal complexes with ct-DNA, viscosity measurements of DNA solutions in the presence of increasing concentrations of the complexes were caried out.

Intercalation binding manifested by insertion of the compound molecule between the base pairs of DNA, causing separation between base pairs at the intercalation site to some degree and an increase in helix length. As a result, the viscosity increased. Other modes of interaction, such as groove binding and electrostatic interactions can bend or deform the DNA helix, shortening its length. This may cause a decrease in DNA viscosity [31].

**Figure 6:**
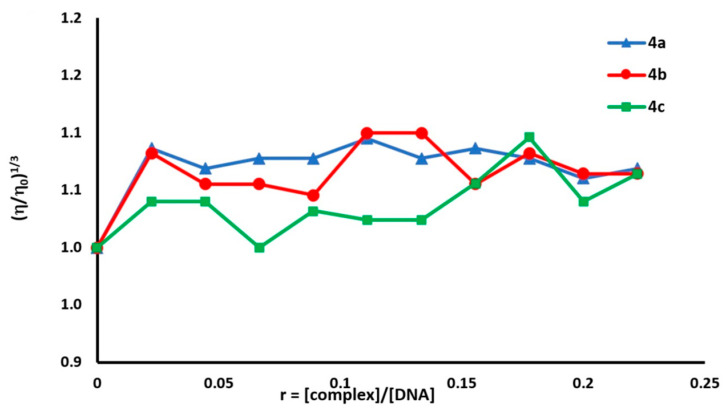
Relative viscosity of DNA in the buffer solution in the presence of studied complexes **4a**–**c** under the condition of increasing the concentration ratio [complex]/[DNA]; [DNA] = 100 µM, [complex] = 0–22 µM.

The effect of adding test compounds **4a**–**c** on the relative specific viscosity of DNA is shown in Figure 6. There was an insignificant change in the viscosity of DNA. As can be seen in the graph, the viscosity of the DNA solution increased only slightly with increasing amounts of complexes **4a**–**c**. Thus, the results indicated that the binding mode of the complexes may be predominantly groove binding. A similar effect on the viscosity of DNA was found in the other copper(II) complexes derived from an asymmetric bidentate Schiff base. Their groove binding mode was consistent with molecular docking results [40].

### 2.5. DNA Cleavage

The ability of metal complexes to bind and cleave DNA has been observed for many copper complexes. The cleavage ability of copper complexes can be determined by the standard gel electrophoresis method [41] and cleavage can occur through multiple pathways. One of them is the hydrolytic mechanism, but in situ hydroxyl radical formation or oxidative cleavage assisted by a redox-active copper center is also possible [42,43,44,45]. We expected the Cu(II) complexes, exhibiting DNA binding affinity and efficient DNA cleavage, to arrest the cell cycle progression of cancer cells and further promote the apoptotic mode of cell death [46,47].

**Figure 7:**
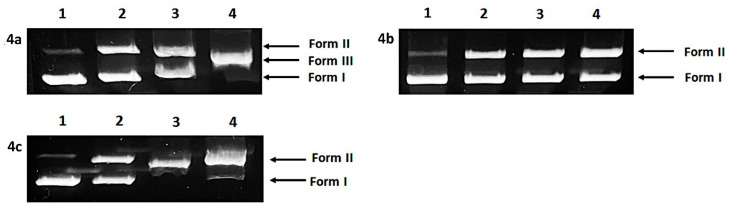
Agarose gel electrophoresis patterns of cleavage of the pBR322 DNA plasmid by copper complexes. **Line 1**—control (undigested DNA plasmid), **Line 2**—DNA plasmid digested with copper complex at concentration of 1 mM, **Line 3**—at concentration of 3 m M, **Line 4**—at concentration of 5 mM. **Form I**—supercoiled plasmid DNA (undigested plasmid), **Form II**—open-circular plasmid DNA (partly digested plasmid), **Form III**—linear plasmid DNA (completely digested plasmid).

The gel electrophoresis separation of the supercoiled DNA plasmid pBR322 induced by prepared copper(II) complexes is shown in Figure 7. One copper(II) complex **4a** completely cleaved plasmid supercoiled DNA into a linear form (Form III), thus demonstrating its highest DNAse activity at a concentration of 5 mM. The other copper(II) complexes (**4b**–**c**) cleaved the plasmid supercoiled form of DNA (Form I) to an open-circular, partly cleaved form (Form II) and showed weaker DNAse activity. The cleavage activity of **4a** and **4c** increased remarkably with increasing concentration.

The DNA cleaving activities were also quantified using the “geneQUANT software” (Cleaver Scientific, UK). Data are expressed as percentage (%) expression of the amount of pDNA that has passed through the effect of the nuclease activity of the copper complex from the uncleaved supercoiled Form I to the cleaved Form II (open-circular pDNA) or Form III (linear pDNA) (Table 5). Each applied sample had a volume of 20 μL, containing 250 ng of pDNA. Therefore, each percentage can also be converted to the mass of pDNA turned to Form II or III. For example, for complex **4a** at a 1 mM concentration, 41.2% of pDNA was cleaved to Form II, which is 103 ng of pDNA, or at a 3 mM concentration, 46.2% corresponds to 115.5 ng of pDNA. Quantification allows us to compare the specific complexes more precisely. As is obvious from the electrophoresis images, complex **4a** has impressive activity at concentration 5 mM (100% cleavage to Form III), but complex **4c** showed almost complete cleavage to Form II already at 3 mM. Complex **4b** with a copper/ligand ratio of 1:2 shows different behavior, with cleaving properties similar to the other complexes at 1 mM (41.5% Form II), but with only a small increase in activity with an increase in concentration. Some aggregation might be present at higher concentrations, keeping the amount of reactive monomer at a lower level.

### 2.6. Interaction of Complexes Studied with BSA

Serum albumin is the most abundant protein present in the blood and its main function is to be a carrier for both exogenous and endogenous substances. The binding affinity between bioactive compounds and serum albumin plays a significant role in the design of novel drugs [48]. Serum albumin can also accumulate in tumor tissue and can serve as a potential macromolecular carrier for the delivery of anticancer agents to solid tumors [49].

The interaction between antitumor drugs and serum albumin can affect the bioavailability and toxicity of these agents. Bovine serum albumin (BSA) represents a well-recognized model for the investigation of intermolecular interactions with drugs due to its high structural similarity to human serum albumin [50].

**Figure 8:**
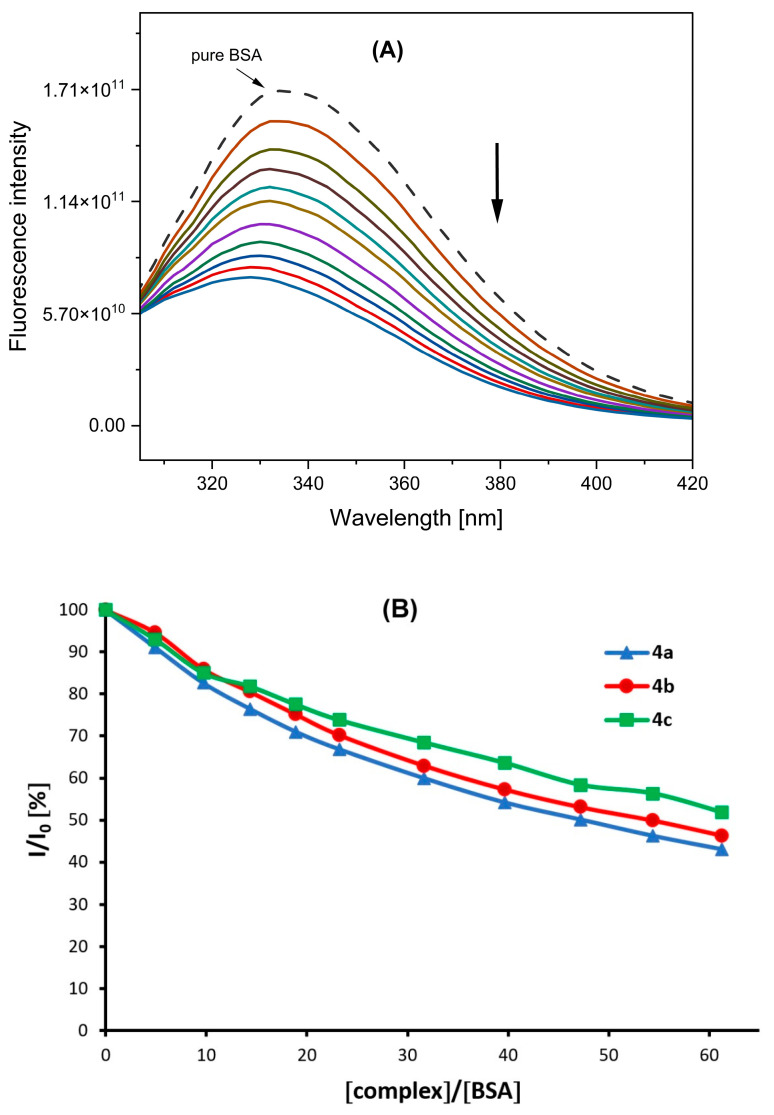
(**A**) Fluorescence emission spectra of BSA in buffer solution in the presence of increasing concentrations of complex **4a**. The arrow indicates changes in fluorescence with increasing amounts of the complex. The concentration of the added complexes ranged from 0 to 1.6 × 10^−2^ mM (the concentration increment was 1.6 × 10^−3^ mM). (**B**) Graphical dependence of relative BSA fluorescence intensity in % at *λ* = 336 nm vs. concentration ratio [complex]/BSA for complexes **4a**–**c**.

The fluorescence emission spectra of BSA show intense fluorescence emission at 336 nm upon excitation at 280 nm. This is due to the presence of two tryptophan residues (Trp134 and Trp212). Fluorescence quenching data were analyzed to obtain various binding parameters for the interaction of complexes **4a**–**c** with BSA. The BSA emission spectra in the presence of complexes are shown in Figure 8A and Figure S4-1.

The graph of relative BSA fluorescence intensity with increasing complex concentration exhibited a significant quenching of fluorescence up to 48–56% (Figure 8B). The maximum emission peak of BSA shifted blue, indicating that hydrophobicity increased, and polarity decreased in the microenvironment of the amino acid residues of BSA [51]. These results may indicate the strong interaction between studied complexes within an albumin tryptophan environment.

The interaction of complexes with serum albumin was characterized by the Stern–Volmer constant *K_sv_* and the quenching constant *k*_q_, which was calculated using the Stern–Volmer equation (Figure S4-2) together with the associate binding constant *K_BSA_* and the number of binding sites per albumin *n* determined from the Scatchard equations (Equation (6), Figure S4-3); the values are summarized in Table 6.

Three mechanisms have been proposed for the quenching of protein fluorescence emission: static, dynamic and a combination of the two mechanisms. The static mechanism requires a drug–protein complex formation at ground state. The dynamic mechanism is based on the collision between drug molecules and proteins. In the combined mechanism, both collisions between the drug and the protein and the formation of a drug–protein complex play their role [15].

The presented values of the quenching constant *k*_q_ of the complexes (10^12^ M^−1^s^−1^) are greater than the 10^10^ M^−1^s^−1^ value which represents a typical value for the maximum scatter collision quenching constant of various quenchers with biopolymers. These large Stern–Volmer values of *k*_q_ for the complexes indicate that quenching is performed through a static quenching mechanism [17,52,53]. In the static quenching mechanism, it is assumed that the binding and free molecules reach an equilibrium, and the binding sites are similar and independent [54].

The *k*_q_ values reported in this work are in good agreement with the values reported in the literature for BSA and other copper(II) Schiff base complexes [32,55,56,57]. The Stern–Volmer curve for all complexes had a linear plot (Figure 8B and Figure S4-2), indicating the presence of static quenching. The formation of a complex in ground state between BSA and Cu complexes was also confirmed by analysis of the UV–Vis absorption spectrum. The protein titration curve for the study complex–BSA interaction was measured preliminarily for complexes **4a** and **4c**. Figure S4-4 (representative spectrum) shows that the UV–Vis absorbance intensity decreased regularly with an increasing concentration of **4c**, indicating that the BSA molecules associated with **4c** and formed a BSA complex.

This again confirmed a static quenching mechanism. The interaction of BSA with metal complexes could result in absorption change of BSA at a 278 nm wavelength, associated with the polarity of the microenvironment around the BSA tyrosine and tryptophan residues. [58] A hyperchromic shift [17,59] or hypochromic effect [60] of BSA absorption intensity caused by interaction of metal complexes with BSA is usually described.

The calculated number of binding sites for albumin—*n* (0,94–1.1623)—could mean that there was one independent class of complex binding sites on albumin. The calculated *K_BSA_* binding constant for the studied complexes (of order 10^4^) is in the optimal range assumed for a serum albumin drug-delivery system (with *K_sv_* within 10^2^–10^8^; binding constant *K_BSA_* within 10^4^–10^6^) [48,61].

### 2.7. Cytotoxicity Based on Mitochondrial Activity

The resazurin-based model that monitors mitochondrial activity is widely used as an indicator of cell viability, proliferation and cytotoxicity. This method is based on the reduction of resazurin to resorufin by mitochondrial enzymes, as carriers of diaphorase activities, like NADPH-dehydrogenase, which are responsible for the transference of electrons from NADPH^+^, H^+^ to resazurin. The reduction of resazurin correlates with the number of live bacterial or mammalian cells or fungi [62]. Furthermore, *S. cerevisiae* is an established model system for eukaryotic organisms [62,63,64]. They have conserved cellular processes with high homology to those of humans. They proliferate rapidly and are even used as a model to study the effects of anticancer agents [65]. The use of *S. cerevisiae* in resazurin redox testing is a good alternative to conventional cell viability testing techniques.

The absorbance values for reduced and oxidized resazurin (measured at 570 nm and 600 nm) obtained are listed in Table 7. The reduction percentage was calculated using the equation Equation (1). The complexes showed excellent cytotoxic activity ranging from 67.1 to 99.0% depending on the concentration of the added complex (Table 7, Figures S5-2 to S5-4). Complex **4c** was most effective (94.8%) already at 1 × 10^−3^ M, followed by **4b** and, surprisingly, **4a** being least active. At higher concentrations, experiments showed cell death above 90%, with similar activities for all three complexes.

### 2.8. Cancer Cell Cytotoxicity by MTT Assay

**Figure 9:**
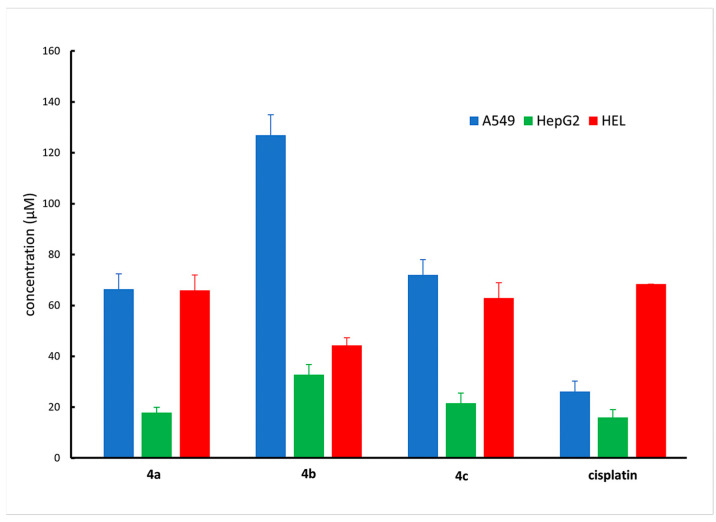
IC_50_ concentrations for cytotoxicity against cancer cells A549 and HepG2 and noncancerous HEL cells.

The cytotoxicity study of the prepared copper(II) complexes was performed using the MTT assay on human cell lines—A549 (adenocarcinoma human alveolar basal epithelial cell line), HepG2 (human liver cancer cell line) and HEL (human lung fibroblast cell line, normal cells). Cytotoxicity was measured at various sample concentration ranges after 24 and 72 h; however, at 24 h the cytotoxicity was low. After 72 h, the activity reached good levels, especially against HepG2 cells. The IC_50_ values were calculated based on the measured data for all prepared complexes and the standard cisplatin (Figure 9 and Table 8). The IC_50_ profiles of all complexes show interesting cytotoxicity against HepG2 with **4a** (17.9 ± 2.6 μM) and **4c** (21.6 ± 4.9 μM) achieving activity comparable to cisplatin (16.0 ± 3.4 μM). All complexes show lower IC_50_ values for HepG2 cancer cells (18–33 μM) than for normal HEL cells (44–66 μM), which is also analogous to cisplatin. A very interesting feature is that the IC50s for HEL in the case of **4a** and **4c** is about 3-times higher than for HepG2. On the other hand, the IC_50_ values for A549 cells were relatively high, with the best IC50 achieved for **4a** at 66.4 ± 6.2 μM. Complex **4b** has the least favorable IC50 for all three cell types.

Cytotoxicity measured against HepG2 cells has an interesting correlation with the pDNA cleavage experiments, where the **4a** and **4c** complexes also showed better cleaving properties with 100% cleavage to Form III at 5 mM for **4a** and 100% cleavage of pDNA to Form II at 3 mM for **4c**. On the other hand, the binding experiments with ct-DNA showed almost identical values for all three complexes, with **4a** being the better EB displacer and **4c** showing the best activities in the absorption titration experiment. The resazurin method used to measure mitochondrial activities showed the main difference of the complexes at 1 mM, with **4c** showing the best results, suggesting cytotoxicity at the mitochondrial level or also on the mitochondrial level [66]. It could also be related to the ROS production which copper, especially Cu(I), can stimulate in cells [67]. Increased oxidative stress in cells caused by the presence of copper can lead to cell death (cuproptosis) by mitochondrial damage, interfering with the tricarboxylic acid cycle by copper binding to specialized sulfur-containing proteins or a loss of mitochondrial membrane potential. This prevents mitochondrial function, can lead to morphological changes within the mitochondria and finally leads to cell death [68,69]. Due to this, it was also interesting to study the ability of the complexes to undergo a single electron absorption or release where copper could change from II to I and back (radical scavenging or SOD mimetic activity). This process can be simulated by radicals capable of giving or taking one electron to or from copper, other metals or even organic molecules. This is a process that also occurs in SOD enzymes, and thus many coordination compounds able to do this are also called SOD mimetics [70].

### 2.9. SOD Mimetic Activity

The radical scavenging ability with respect to the SOD mimetic activity of the prepared complexes was determined by the INT method. This method is based on a competitive reaction with the superoxide anion radical from KO_2_ between the measured compound (complex) and 2-(4-Iodophenyl)-3-(4-nitrophenyl)-5-phenyl-2*H*-tetrazolium chloride (INT). INT reacts with the single electron of O_2_^−^ and produces blue-green formazane detected as light absorption at 500 nm. Complexes with good antiradical activity also react with O_2_^−^, and as a result of this, less formazane is formed. Complexes with very good activity can almost completely inhibit the transfer of electrons on INT.

The first type of antiradical activity measurements involved the determination of inhibition percent values of electron transfer on INT at specific concentrations (Table 9). Two concentrations were selected to achieve good solubility of the complexes (4 and 1 mM for **4a** and **4c**, 2 and 1 mM for **4b**). The complex **4b** dissolved readily at a concentration of 4 mM, but the mixture was very dark, making measurements at 500 nm difficult. Therefore, a less concentrated initial solution was used (2 mM) as the higher concentration. In total, 0.5 mL of the initial solutions was used to prepare sample solutions that also contained INT and KO_2_ solutions and DMSO with an overall volume of 3.5 mL. The resulting concentrations were c_1_ = 0.572 mM, c_1*_ = 0.286 mM and c_2_ = 0.143 mM, respectively. Complexes **4a** and **4c** once again provided high percentages at c_1_ (approximately 96–99%) but the activity dropped below 24% at c_2_, while complex **4b** maintained mediocre activity at both concentrations. It seems that the concentration increase had only a small effect on the activity of **4b**; something similar was also observed in the DNA cleavage study for complex **4b**. The complexes were compared with the model compound cystamine, which was used as a standard for superoxide antiradical scavenging due to the presence of a free thionyl group, which is also present in glutathione.

To improve the comparability of SOD mimetic activity, IC_50_ concentrations were determined by performing a series of measurements with decreasing concentration. IC_50_ values were calculated from the trend function equation as the concentration with 50% inhibition of electron transfer on INT (Table 10). The IC_50_ values confirmed that complex **4c** was the most effective radical scavenger, followed by **4a**. The least active was complex **4b**, for which 50% inhibition was reached in the >1 mM range, the same as cystamine.

Comparing the results from the radical scavenging measurements with the cytotoxicity showed certain common features. Antimitochondrial cytotoxicity (resazurin method) showed high activity, especially for complex **4c** (above 94%), which also showed the highest DNA cleavage at 3 mM. The SOD-IC50 value was lowest for complex **4a**, which also showed the best IC50 values in the MTT cytotoxicity test against both cancer cell types, but was also the only complex capable of performing double-strand cleavage on pDNA with 100% efficiency, but at 5 mM. It would seem that while **4c** also relies on mitochondrial toxicity, **4a** might make use of some ROS generation at higher concentrations.

## 3. Materials and Methods

### 3.1. Instrumentation and Materials Used in Synthesis

All chemicals for syntheses were reagent grade and were used as received from Sigma-Aldrich (Merck, Taufkirchen, Germany), Acros Organics (Thermo Fisher Scientific Inc., Waltham, MA, USA), and Alfa-Aesar (Thermo Fisher Scientific, Waltham, MA, USA), except for methanol which was dried using predrying with calcium oxide and reflux with magnesium activated with iodine and distillation.

All NMR spectra were measured on a Varian Gemini 2000 spectrometer (Varian Medical Systems, Palo Alto, CA, USA) at working frequencies of 300 MHz for ^1^H NMR and 75 MHz for ^13^C NMR. Spectra were measured in CDCl_3_, using TMS as an internal standard. For NMR signal assignment, the numbering always starts on the phenolic ring with 1 on the carbon where the iminomethyl is located (1–6, Ar), followed by the two carbons in the linker between the aromatic rings (7 and 8), the methoxyphenyl has its own numbering (1′–6′, Ar′) and the carbon of the methoxy group is numbered 9. Infrared spectra were recorded on a Nicolet 6700 FT-IR spectrophotometer (Thermo Fisher Scientific, Waltham, MA, USA) in the range of 500–4000 cm^−1^ and the samples were in the oil or solid state. Elemental analysis was measured with the Flash 2000 CHNS-O Analyser (Thermo Fisher Scientific, Waltham, MA, USA). UV–Vis spectra were measured in DMSO using a Genesys 10S UV–Vis spectrometer (Thermo Fisher Scientific, Waltham, MA, USA). The HRMS spectra for stability studies were measured by Orbitrap LTQ XL (Thermo Fisher Scientific, Waltham, MA, USA).

### 3.2. General Procedure for Synthesis of the Schiff Base Ligands (***3***)

In a two-neck flask with a reflux condenser, a mixture of 2-, 3- or 4-methoxybenzylamine (**2a**–**c**) (1.31 g, 9.58 mmol, 1 mol equiv) in 20 mL of dry methanol was prepared. The mixture of salicylaldehyde (**1**) (1.17 g, 9.58 mmol, 1 mol equiv) in 5 mL of dry methanol was added via a septum and the whole reaction mixture was heated with reflux while monitored by TLC (hexane/ethyl acetate = 19:1). After 1–2 h, the reaction came to completion and the mixture was cooled down to room temperature. Methanol was evaporated, which resulted in a deep yellow oil. The oil was washed three times with diethyl ether (20 mL) and dried at low pressure to obtain products **3a**–**c** as yellow syrupy oils.

**2-[(2-Methoxy-benzylimino)-methyl]-phenol (Sal-2-MeOBn, 3a)** was obtained as yellow oil in 2.17 g (93%). ^1^H NMR (300 MHz, CDCl_3_, ppm) δ: 8.40 (s, 1H, C7-H), 7.23–7.33 (m, 4H, C4-H, C6-H, C4′-H a C6′-H), 6.83–6.97 (m, 4H, C3-H, C5-H, C3′-H and C5′-H), 4.80 (s, 2H, C8-H_2_), 3.85 (s, 3H, C9-H_3_). ^13^C NMR (75 MHz, CDCl_3_, ppm) δ: 165.5 (C7), 161.4 (C2′), 157.1 (C2), 132.1 (C4), 131.3 a 129.1 (C6 a C6′), 128.4 (C4′), 126.3 (C1′), 120.6 (C5 or C5′), 119.0 (C1), 118.4 (C5 or C5′), 117.1 (C3), 110.3 (C3′), 57.7 (C9), 55.3 (C8). Elemental analysis calculated for C_15_H_15_NO_2_ (M_r_ = 241.29): C 74.67; H 6.27; N 5.81%; measured: C 74.38; H 6.15; N 5.72%. IR (oil, cm^−1^): 2962 (w), 2843 (w), 1633 (s, N=C), 1601 (w), 1578 (m), 1492 (s), 1457 (s), 1444 (m), 1428 (m), 1412 (w), 1279 (s), 1239 (s), 1212 (w), 1199 (w), 1175 (w), 1165 (w), 1149 (w), 1110 (m), 1061 (s), 1022 (s), 996 (w), 963 (w), 941 (w), 844 (m), 755 (s), 735 (m), 656 (m).

**2-[(3-Methoxy-benzylimino)-methyl]-phenol (Sal-3-MeOBn, 3b)** was prepared as yellow oil in 2.31 g (99%). ^1^H NMR (300 MHz, CDCl_3_, ppm) δ: 8.42 (s, 1H, C7-H), 7.24–7.34 (m, 3H, C4-H, C6-H a C5′-H), 6.81–6.98 (m, 5H, C3-H, C5-H, C2′-H, C4′-H, C6′-H), 4.77 (s, 2H, C8-H_2_). 3.80 (s, 3H, C9-H_3_). ^13^C NMR (75 MHz, CDCl_3_, ppm) δ: 165.6 (C7), 161.1 (C3′), 159.8 (C2), 139.7 (C1′), 132.3 (C4), 131.4 (C6), 129.7 (C5′), 120.0 (C1), 118.8 and 118.6 (C5 and C6′), 117.0 (C3), 113.4 (C2′), 112.7 (C4′), 63.1 (C9), 55.2 (C8). Elemental analysis calculated for C_15_H_15_NO_2_ (M_r_ = 241.29): C 74.67; H 6.27; N 5.81%; measured: C 74.56; H 6.21; N 5.75%. IR (oil, cm^−1^): 2997 (w), 2907 (w), 2831 (m), 1634 (s, N=C), 1601 (s), 1582 (w), 1491 (s), 1461 (s), 1425 (s), 1221 (w), 1205 (w), 1154 (s), 1115 (m), 1068 (m), 1044 (s), 997 (w), 967 (m), 937 (w), 909 (m), 851 (s), 839 (m), 785 (s), 754 (s), 747 (m), 732 (w), 692 (s), 656 (m), 626 (w).

**2-[(4-Methoxy-benzylimino)-methyl]-phenol (Sal-4-MeOBn, 3c)** was obtained as a yellow oil in 2.24 g (97%). ^1^H NMR (300 MHz, CDCl_3_, ppm) δ: 8.38 (s, 1H, C7-H), 7.29 (ddd, 1H, *J*(3,4) = 8.2 Hz, *J*(4,5) = 7.3 Hz, *J*(4,6) = 1.6 Hz, C4-H), 7.19–7.26 (m, 3H, C6, C2′, C6′), 6.95 (dd, 1H, *J*(3,4) = 8.2 Hz, *J*(3,5) = 1.0 Hz, C3-H), 6.84–6.90 (m, 3H, C5, C3′, C5′), 4.72 (s, 2H, C8-H_2_), 3.79 (s, 3H, C9-H_3_). ^13^C NMR (75 MHz, CDCl_3_, ppm) δ: 165.2 (C7), 161.1 (C4′), 158.9 (C2), 132.4 (C4), 131.3 (C6), 130.2 (C1′), 129.0 (C2′ a C6′), 118.8 (C1), 118.5 (C5), 117.0 (C3), 114.1 (C3′ and C5′), 62.5 (C9), 55.3 (C8). Elemental analysis calculated for C_15_H_15_NO_2_ (M_r_ = 241.29): C 74.67; H 6.27; N 5.81%; measured: C 74.54; H 6.31; N 5.78%. IR (oil, cm^−1^): 3009 (w), 2900 (w), 2838 (w), 1629 (s, N=C), 1609 (s), 1585 (m), 1512 (s), 1464 (w), 1443 (w), 1421 (w), 1375 (w), 1327 (w), 1301 (m), 1283 (s), 1247 (s), 1215 (w), 1183 (s), 1150 (w), 1119 (w), 1040 (m), 1031 (s), 989 (w), 951 (w), 864 (w), 848 (s), 837 (s), 825 (s), 782 (w), 764 (s), 738 (w), 718 (w).

### 3.3. General Procedure of Synthesis of Schiff Base Ligand Complexes (***4***)

The solution of CuCl_2_.2H_2_O (141 mg, 0.829 mmol, 1 mol equiv) in 5 mL of methanol was added to the solution of the Schiff base **3** (200 mg, 0.829 mmol, 1 mol equiv) in 10 mL of methanol at room temperature. This caused a dramatic color change and, after 1–2 days, the product precipitated in the form of a dark green powder or crystals. The product was isolated by vacuum filtration and washed with a small amount of methanol and diethyl ether.

**[Cu_2_ (Sal-2-MeOBn)_2_ Cl_2_] (4a)** was isolated as crystals in 125 mg (44.5%). Elemental analysis calculated for C_30_H_28_Cl_2_Cu_2_N_2_O_4_ (M_r_ = 678.55): C 53.10; H 4.16; N 4.13%; measured: C 53.42; H 4.17; N 4.22%. IR (solid, cm^−1^): 3007 (w), 2962 (w), 2934 (w), 2832 (w), 1626 (s, C=N), 1596 (s), 1558 (s), 1495 (m), 1476 (m), 1463 (w), 1432 (m), 1411 (w), 1347 (m), 1325 (m), 1276 (s), 1254 (s), 1224 (m), 1162 (m), 1122 (s), 1049 (m), 1030 (s), 990 (w), 954 (w), 897 (m), 838 (m), 763 (s), 744 (s), 658 (m), 613 (m). UV–Vis (nm): 278, 298, 370, 712.

**[Cu (Sal-3-MeOBn)_2_] (4b**) was obtained as a powder in 102 mg (45%). Elemental analysis calculated for C_30_H_28_CuN_2_O_4_ (M_r_ = 544.10): C 66.22; H 5.19; N 5.15%; measured: C 66.00; H 5.22; N 5.23%. IR (solid, cm^−1^): 3087 (w), 3026 (w), 2999 (w), 2960 (w), 2936 (w), 2837 (w), 1621 (s, C=N), 1614 (s), 1584 (m), 1542(s), 1489 (m), 1473 (s), 1451 (m), 1437 (s), 1399 (m), 1350 (m), 1328 (s), 1292 (w), 1259 (s), 1230 (w), 1201 (m), 1146 (s), 1127 (w), 1045 (s), 989 (m), 911 (m), 884 (w), 873 (m), 853 (w), 809 (w), 780 (m), 750 (s), 737 (m), 697 (s), 612 (m). UV–Vis (nm): 278, 300, 365, 695. MS (pos., aq. DMSO) m/z: 544.1550 (theor. 544.1554).

**[Cu_2_ (Sal-4-MeOBn)_2_ Cl_2_] (4c)** was isolated as a powder in 107 mg (38%). Elemental analysis calculated for C_30_H_28_Cl_2_Cu_2_N_2_O_4_ (M_r_ = 678.55): C 53.10; H 4.16; N 4.13%; measured: C 53.44; H 4.19; N 4.19%. IR (solid, cm^−1^): 3064 (w), 3000 (w), 2925 (w), 2834 (w), 1629 (s, C=N), 1599 (s), 1559 (s), 1507 (s), 1475 (m), 1463 (w), 1444 (m), 1407 (w), 1334 (w), 1299 (w), 1276 (s), 1249 (s), 1216 (m), 1172 (m), 1158 (m), 1112 (w), 1044 (w), 1030 (s), 997 (w), 957 (w), 897 (m), 848 (m), 831 (m), 795 (w), 768 (s), 715 (w), 658 (m), 626 (w). UV–Vis (nm): 278, 297, 361, 709.

### 3.4. Stability Measurements

The stability of the complexes was studied by using UV–Vis. All prepared complexes were sufficiently soluble in all measurements used. The solutions of the complexes in DMSO and citrate buffer solution were studied over up to 72 h (Figures S0-2–S0-7 in Supplementary Materials). We conducted several more UV–Vis spectroscopic studies, where we proved complex formation and that the complexes are the same in solid form and right after dissolution in DMSO and one hour later (Figure S0-8). We could see no changes for complexes **4a**–**c** and the spectra remained the same as in the long-term study.

### 3.5. X-Ray Structure Analysis

A STOE STADIVARI diffractometer (STOE Corporation, Chicago, IL, USA) was used to measure the diffraction data of complex **4a**. The diffractometer was equipped with Dectris Pilatus 300 K detector, with a Genix3D Cu HF source (Cu-K_α_, λ = 1.54186 Å) at 100 K using a nitrogen gas open-flow cooler Cobra (Oxford Cryosystems, Oxford, UK). Data reduction was performed using the X-Area software package (version 1.84, STOE & Cie GmbH, Darmstadt, Germany). The crystal structure of complex **4a** was solved in the OLEX2 software (version 1.5) [71] using the SHELXT-2015 program via Intrinsic Phasing [72] and further refined with SHELXL-2015 by the least-squares procedure on F^2^ [73]. All non-hydrogen atoms were refined with anisotropic thermal parameters. Hydrogen atom positions were optimized under the constraints of bonding to their parent atoms, an aliphatic C–H bond length of 0.99 Å, with an aromatic C–H bond length of 0.95 Å, a methyl group C–H bond length of 0.98Å and a C–H bond (on the imine carbon) length of 0.95Å. The temperature factors of hydrogen atoms were U_iso_(H) = 1.2 U_eq_(C), for the methyl group U_iso_(H) = 1.5 U_eq_(C) and the water molecule U_iso_(H) = 1.5 U_eq_(O). For molecular images, the DIAMOND program (version 2.1e) [74] and the MERCURY (version 3.8) program [75] were used.

Accession Codes

The crystallographic data used in this paper can be accessed as CCDC 2367651 via www.ccdc.cam.ac.uk/data_request/cif, by e-mailing data_request@ccdc.cam.ac.uk or by contacting The Cambridge Crystallographic Data Centre, 12 Union Road, Cambridge CB2 1EZ, UK; fax: +44 1223 336033.

### 3.6. EPR Studies

EPR spectra were recorded using an X-band CW EPR spectrometer Bruker EMX Plus (Bruker, Germany) with a standard TE_102_ (ER 4102 ST) rectangular resonator cavity (Bruker, Germany). For spectra acquisition and processing, the software package Bruker Acquisition and WinEPR (version 2.11, Bruker, Bremen, Germany) was used. Solid-state spectra with a linear slope are shown as baseline corrected. The microwave power of 20 mW was tested and considered far below saturation for all samples. All CW EPR spectra were recorded using 100 kHz field modulation with 5 G (0.5 mT) amplitude. Due to the expectation of ZFS (zero field splitting), spectra were recorded within the entire available field range of our spectrometer (0–1500 mT). For solid-state measurements, a small amount of solid state powder was placed into a thin-wall quartz EPR tube (internal diameter 3 mm). Low-temperature measurements were performed using a special Dewar finger inset inserted directly into the EPR resonator cavity, enabling the direct placement of the sample into liquid nitrogen surroundings. Room temperature was 300 K. Spectra were simulated using the Easyspin simulation program (version 6.0.10) [76,77] with Simultispin extension [78]. The integral intensity of the signal in spectra is not directly related just to the number of spins in the sample because of the complex nature of EPR signal intensity, which is dependent on many factors that influence the sensitivity of detection. Quantity was not a goal within this measurement and would require another different experimental approach.

### 3.7. DNA Cleavage Assay

The ability of copper(II) complexes to cleave DNA was examined by following the conversion of supercoiled plasmid DNA (Form I) to the open-circular DNA (Form II) and/or linear DNA (forms using agarose gel electrophoresis to separate the cleavage products [79,80].

In general, 250 ng of the DNA plasmid pBBR 322 (in 25 mM TRIS-HCl buffer solution, pH 7.5) was treated with different concentrations (1 mM, 3 mM and 5 mM) of the copper(II) complex (dissolved in DMSO) for 3 h at 37 °C. After that, each reaction was quenched by adding 4 μL of a Smart Glow Loading Dye^®^ (loading buffer solution with 0.01% Safe Green dye, 50% glycerol and 250 mM EDTA, pH 8.0) and the sample subjected to electrophoresis (MyGel Mini electrophoresis system^®^, Accuris, Edison, NJ, USA) on a 0.9% agarose gel containing TAE buffer (40 mM TRIS, 20 mM acetic acid and 1 mM EDTA) at a voltage of 55 V/40 mA for about 1.5 h. As a “running buffer”, TAE buffer (40 mM TRIS, 20 mM acetic acid and 1 mM EDTA) was used. The resulting gels were visualized with UV light and scanned by a mobile device, an Apple iPhone 11 cam 26 mm f1.8. DNA cleaving activities were quantified using the “geneQUANT software” (version 4.3.9, Cleaver Scientific, Rugby, UK).

### 3.8. DNA Binding Study by Absorption Titration

The interactions of prepared complexes **4a–c** with ct-DNA have been studied by UV–Vis monitored titration. The ct-DNA solution was prepared by dissolving 6 mg of ct-DNA in 5 mL of citrate buffer (composed of 15 mM sodium citrate and 150 mM sodium chloride mixture solution, at pH = 7.0). The concentration of ct-DNA was then determined by UV–Vis spectroscopy with the molar absorption coefficient of ct-DNA at 260 nm (6600 M^−1^cm^−1^). The studied complexes were dissolved in DMSO, from which working solutions were prepared by dilution using citrate buffer solution. Increasing concentrations of ct-DNA were then added to a buffer/DMSO solution (<1% DMSO) of the corresponding complex (1 × 10^−4^ mol⋅ml^−1^) [81].

For the values of the binding strength of the interaction between the complex and the DNA molecule, binding constants K_b_ were calculated by the ratio of slope to the y intercept in the plots [DNA]/(ε_A_-ε_f_) compared to [DNA], according to the Wolfe–Shimer equation (Equation (1)):

(1) $$\frac{\left[DNA\right]}{\left(\varepsilon_{A}-\varepsilon_{f}\right)}=\frac{\left[DNA\right]}{\left(\varepsilon_{b}-\varepsilon_{f}\right)}+\frac{1}{K_{b}(\varepsilon_{b}-\varepsilon_{f})}$$

where [DNA] is the concentration of DNA in base pairs, *ε_f_* is the extinction coefficient of the free complex at the corresponding λ_max_, *ε_A_* = A_obsd_/[compound] and *ε_b_* is the extinction coefficient of the complex in the fully bound form.

Control measurements with DMSO were carried out with no changes in the ct-DNA spectra observed.

### 3.9. EB–DNA Displacement Method

Competitive-binding experiments to determine the displacement of the intercalator ethidium bromide (EB) from the EB–DNA adduct were conducted by fluorescence spectroscopy. To prepare the EB–DNA adduct, 20 μM EB and 54 μM ct-DNA were added to citrate buffer (sodium citrate at 15 mM and sodium chloride at 150 mM at pH = 7.0) [82]. The addition of increasing concentrations of complexes **4a**–**c** (c = 1 mM) to a solution of the EB–DNA complex corresponded to the intercalating effect of the complexes. The concentration of the added complexes ranged from 0 to 0.2 mM. Fluorescence emission spectra were recorded in the range of 550–800 nm with an excitation wavelength of 515 nm. The complexes did not show any fluorescence emission bands in their own solution or in the presence of ct-DNA or EB. Quenching of the EB–DNA emission band by the compounds **4a**–**c** was calculated using the Stern–Volmer equation (Equations (2)–(4)).

The extent of the inner-filter effect can be roughly estimated with the following equation:

(2) $$I_{corr}=I_{meas}\times 10^{\frac{{\varepsilon (\lambda }_{exc})cd}{2}}\times 10^{\frac{{\varepsilon (\lambda }_{em})cd}{2}}$$

where *I_corr_* is the corrected intensity, *I_meas_* is the measured intensity, c is the quencher concentration, d is the width of the cuvette (1 cm) and *ε_(λexc)_* and *ε_(λem)_* are the excitation coefficients of the quencher at the excitation and the emission wavelength calculated from the UV–Vis spectra of the complexes.

The quenching of the EB–DNA emission band by compounds was calculated via the Stern–Volmer equation:

(3) $$\frac{Io}{I}=1+k_{q}\tau_{0}\left[Q\right]=1+K_{SV}\left[Q\right]$$

where *Io* = the initial fluorescence intensity of the EB–DNA adduct, and *I* is the fluorescence intensity of the EB–DNA adduct after the addition of the quencher (studied complexes), *k_q_* = the quenching rate constant, *K_SV_* = the Stern–Volmer constant, *τ_o_* = the average fluorescence lifetime of the EB–DNA adduct (23 × 10^−9^ s), [Q] = the quencher concentration, respectively, *K_SV_* (in M^−1^) can be obtained by the slope of the Stern–Volmer plot and *k_q_* (in M^−1^ s^−1^) can be calculated from Equation (4):

(4) $$K_{SV}=k_{q}\tau_{0}$$

### 3.10. Viscosimetric Studies

Changes in the viscosity of the DNA solution (0.1 mM) were measured in the presence of increasing concentrations of the compounds in citrate buffer solution (15 mM of sodium citrate and 150 mM of sodium chloride at pH = 7.0) at a constant 25 °C. The measurements were performed using the ALPHA L Fungilab rotational viscometer (IKA, Staufen, Germany) at 60 rpm, equipped with an 18 mL ELVAS spindle. The relationship between the relative solution viscosity (η/η_0_) and DNA length (I/I_0_) can be seen in Equation (5), where η and η_0_ are the viscosities of DNA in the presence and absence of the studied complex.

(5) $${\left(\frac{\eta }{\eta_{0}}\right)}^{1/3}=\frac{I}{I_{0}}$$

### 3.11. Bovine Serum Albumin Binding Studies

The BSA binding of complexes **4a**–**c** was studied by tryptophan fluorescence emission quenching experiments using BSA (3 μM) in citrate buffer solution (containing 15 mM trisodium citrate and 150 mM sodium chloride at pH 7.0). The emission intensity quenching of BSA tryptophan residues at 336 nm was measured by increasing the concentration of the quencher complexes **4a**–**c** [83]. The concentration of the added complexes ranged from 0 to 0.2 mM. The fluorescence emission spectra were recorded in the 300–420 nm range and with an excitation wavelength of 280 nm. The values of the Stern–Volmer constant *K_SV_* (in M^−1^), the BSA binding constant *K* (in M^−1^) for the compound–BSA interaction and the BSA quenching constant *k_q_* (in M^−1^ s^−1^) have been calculated from the Stern–Volmer (Equations (2)–(4)). and Scatchard equations (Equation (6)):

(6) $$\frac{\Delta I/Io}{\left[Q\right]}=nK-K\frac{\Delta I}{Io}$$

The BSA binding constant *K* (in M^−1^) can be calculated from the slope of the Scatchard plots, and the number of binding sites per albumin (*n*) is given by the ratio of the y intercept to the slope.

Also, to prevent the undesirable effects of dilution on peak intensity in these experiments, a correction for working standard solution dilution was completed.

### 3.12. Resazurin Cytotoxicity Assay

**Scheme 2:**
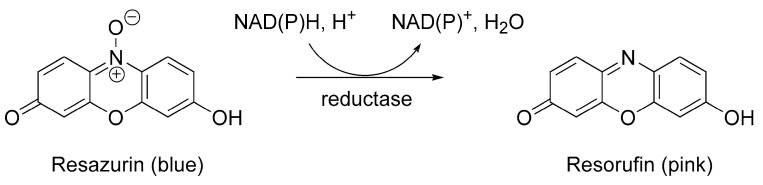
Conversion of resazurin to resorufin in the mitochondria of living cells.

The assay is based on the ability of living cells to reduce blue resazurin (7-hydroxy-3*H*-phenoxazin-3-one 10-oxide) to pink resorufin (7-hydroxy-3*H*-phenoxazin-3-one) which is measurable by the UV–Vis spectrophotometric or fluorometric method (AlamarBlue^TM^). Living cells have active *N*-methylphenazinium methosulfate that catalyzes the redox reaction in mitochondria of NADH and blue resazurin to produce pink resorufin (Scheme 2) [83,84,85,86]. This test is used as an oxidation–reduction indicator in cell viability assays for yeasts and mammalian cells. Reduction of the dye by viable cells decreases the amount of the oxidized form (blue) and, at the same time, increases the amount of its fluorescent intermediate (pink), indicating the degree of cytotoxicity caused by the test material [83,84]. The resazurin-based assay shows excellent correlation with reference viability assays such as formazane-based assays (MTT/XTT) and tritiated thymidine-based techniques [62].

*S. cerevisiae* cells (CCY 21-4-64; Libáň, Czech Republic) were cultured at 28 °C/24h on Saburaud’s dextrose agar with chloramphenicol (Merck, Boston, MA, USA). Subsequently, after 24 h, *S. cerevisiae* cells were inoculated into sterile liquid YPD medium (Sigma–Aldrich, St. Louis, MO, USA) and cultivation continued at 28 °C/24 h. In the exponential growth phase, cells were centrifuged (8.000 g/3 min), washed twice with sterile physiological solution (0.9% NaCl) and pipetted at a concentration of 1 *×* 10^9^ cells/mL in sterile microtubes (modified procedure from Bitacura, 2018) [62]. Sterile physiological solution and copper complexes at concentrations of 1 *×* 10^−3^ M, 3 *×* 10^−3^ M and 5 *×* 10^−3^ M were added to the centrifuged biomass to a total volume of 1 mL and individual samples were cultured in an incubator at 28 °C/3 h. After 3 h, 0.1 mL of Resazurin_OX_ (in vitro toxicology assay kit based on resazurin, Sigma, USA) was added to each sample and the samples were allowed to culture for 2 h. After incubation, the biomass in the samples was separated from the supernatant by centrifugation (8000 g/5 min). The separated solution was diluted in a ratio of 1:1 with physiological solution and subjected to the measurement of UV spectra by the spectrophotometric method according to the recommended protocol of the kit manufacturer (Sigma, USA) on a GENESYS 10S UV–Vis spectrophotometer (Thermo Scientific, USA) in the range of 500–800 nm, using a 1 cm long cuvette.

Cells’ innate metabolic was measured by using an established formula for the reduction percentage. The reduction percentage was calculated using the following formula:

(7) $$Red(\%)=\frac{{Ɛ}_{OX\_600\;nm}\times A_{570\;nm\_sample}-{Ɛ}_{OX\_570\;nm}\times A_{600\;nm\_sample}}{{Ɛ}_{RED\_570\;nm}\times A_{600\;nm\_C-}-{Ɛ}_{RED\_600\;nm}\times A_{570\;nm\_C-}}\times 100$$

**Table 10:** Molar extinction coefficients for reduced and oxidized resazurin [87].

**Wavelength**	**Reduced Resazurin (Resorufin)** **Ɛ_RED_**	**Oxidized Resazurin** **Ɛ** **_OX_**
570 nm	155,677	80,586
600 nm	14,652	117,216

### 3.13. MTT Cytotoxicity Assay

The cytotoxicity of the prepared copper(II) complexes in human cell lines was evaluated using the 3-(4,5-dimethylthiazol-2-yl)-2,5-diphenyltetrazolium bromide (MTT) assay following the protocol by Mosmann. Briefly, A549 (human lung epithelial cells, carcinoma), HepG2 (human liver epithelial cells, carcinoma) and HEL (human lung fibroblast cells, normal) cells were seeded in plastic 96-well plates at a density of 1.5–2 × 10^4^ cells per well. The cells were treated for 72 h with complexes **4a**–**c** and cisplatin dissolved in DMSO and incubated at 37 °C in a 5% CO_2_ atmosphere. After the treatment, the samples were washed with phosphate-buffered saline (PBS), followed by 4 h of incubation with 1 mg/mL of MTT. Subsequently, the MTT solution was removed, and the formed formazane solids were dissolved in dimethyl sulfoxide for 30 min. The photometric evaluation (at 540 nm and 690 nm) was carried out using an xMark™ Microplate Absorbance Spectrophotometer (Bio-Rad, Hercules, CA, USA) [88].

Cell lines

A549 (ATCC^®^ CCL-185), HepG2 (ATCC^®^ HB-8065) and HEL299 (ATCC^®^ CCL-137) cells were obtained from Lambda Life (Bratislava, Slovakia). A549 cells were kept in Dulbecco’s Modified Eagle’s Medium (DMEM) supplemented with 10% fetal bovine serum (FBS) and 1% antibiotics (penicillin and streptomycin). HepG2 cells were kept in Williams medium supplemented with 10% fetal bovine serum (FBS) and 1% antibiotics (penicillin and streptomycin). HEL299 cells were kept in Minimum Essential Medium (MEM) supplemented with 10% fetal bovine serum (FBS), 1% antibiotics (penicillin and streptomycin) and 1% non-essential amino acids (NEAA). The cells were cultured in a humidified atmosphere of 5% CO_2_ at 37 °C. Once the cells reached approximately 80% confluence, they were harvested and subcultured after trypsin treatment.

### 3.14. Radical Scavenging Assay

The method is based on the agent 2-(4-Iodophenyl)-3-(4-nitrophenyl)-5-phenyl-2H-tetrazolium chloride (INT), able to scavenge a superoxide radical anion from KO_2_. The reaction produces a blue-green formazane, which is detected as light absorption at 500 nm. The radical scavengers (measured complexes) compete with INT for radicals and reduce the formation of formazane and light absorption at 500 nm. Antiradical activity was measured on a Synergy HT BioTek spectrophotometer. DMSO was used as the solvent for the complex and KO_2_ (saturated solution). The INT initial solution (4 M) was prepared in a sodium tetraborate buffer solution with pH adjusted to 7.1 with HCl [24,25].

For each measurement, an initial solution of the sample (0.5 mL) of a specific concentration (4–0.1 mM) was prepared in DMSO. Due to the addition of INT (0.5 mL) and KO_2_ (0.5 mL) solutions and additional DMSO (2 mL), the concentrations of the measured complexes decreased seven times (3.5 mL of total mixture). Each result was the average value of three parallel measurements expressed with a standard deviation (more information in the Supplementary Materials). For the determination of SOD-IC50, several measurements with decreasing sample concentration were performed. The percentage of inhibition had a decreasing tendency with decreasing concentration. A linear trend function of the percentage of inhibition and negative logarithm of concentration was used to calculate the SOD-IC_50_ value as the concentration with 50% inhibition of INT–formazane formation.

## 4. Conclusions

Schiff base ligands (**3a**–**c**) derived from salicylaldehyde and 2-, 3- and 4-methoxybenzylamines were prepared. These ligands were used to prepare three copper(II) complexes (**4a**–**c**) from which **4a** could also be studied with X-ray structure analysis. The analysis of complex **4a** revealed a dimeric structure in the solid state, and we also expect a similar structure in complex **4c**. In complex **4b** with a ratio of 1:2, we expect both ligands to bind in bidentate style (N,O) on the same copper center.

The prepared complexes were used in DNA binding studies by absorption titration and EB–DNA displacement methods. The EB–DNA method did not reveal significant differences between the complexes; this is probably due to the similar planar shape of the Schiff base. The best binding properties were observed for *ortho-* and *para*-substituted derivatives (**4a**, **4c**). The BSA binding studies also revealed similar results, with **4c** being the best binder. However, all complexes had a binding constant range for reversible BSA binding. The DNA cleavage studies (pDNA model) showed up to 100% cleavage to Form II at 3 mM for complex **4c** and to Form III at 5 mM for complex **4a**. The cytotoxicity of the complexes was studied in a cell survival model monitoring mitochondrial activity of *S. cerevisiea* (resazurin model) and the MTT assay on cancer cell lines (HepG2 and A549) versus HEL fibroblast cells. The resazurin model revealed a high activity of **4c** with around 95% cell death at 1 mM concentrations. The MTT assay uncovered good activities of **4a** and **4c** against HepG2 cells, comparable with cisplatin activity. These two complexes provided an IC50 more than three-times higher for HEL noncancer cells than for HepG2 cells. The position of the methoxy group on the ligands played a role in the activities of the copper(II) complexes. Complex **4a** with *ortho* position of the methoxy group was highly active in most of our studies, closely followed by **4c** with *para* position. The exception was cytotoxicity based on mitochondrial activity, where **4c** was much more active.

Overall, complexes **4a** and **4c** show a combination of activities that all contribute to the overall cytotoxicity. The DNA binding and cleavage, the halting of mitochondrial activity and the oxidation–reduction reaction provided by the copper center all play a role in this effect.

## Figures and Tables

**Table 1:** Crystal data and structure refinement for complex **4a**.

Empirical Formula	C_30_H_28_Cl_2_Cu_2_N_2_O_4_
Temperature [K]	100(1)
Wavelength [Å]	1.54186
Crystal system	Monoclinic
Space group	P2_1_/c
Unit cell dimensions [Å], [°]	a = 9.2198(1) b = 17.4213(3) c = 17.4576(2) β = 92.412(1)
Formula weight	678.52
Volume [Å^3^]	2801.57(7)
Z/Calculated density [Mg/m^3^]	4/1.609
Absorption coeff. [mm^−1^]	3.955
F(000)	1384.0
Crystal size [mm]	0.2 × 0.04 × 0.02
Theta range for data collection	7.172 to 143.116°
Index ranges	−11 ≤ h ≤ 5 −20 ≤ k ≤ 21 −21 ≤ l ≤ 21
Reflections collected/ Independent reflections	83,351/5393 [R_(int)_ = 0.0325, R_σ_ = 0.0166]
Refinement method	Full-matrix least-squares on F^2^
Data/restraints/parameters	5393/0/363
Goodness-of-fit on F^2^	1.088
Final R indices [I > 2σ(I)]	R_1_ = 0.0259, wR_2_ = 0.0775
R indices (all data)	R_1_ = 0.0303, wR_2_ = 0.0791
Largest diff. peak and hole [e.Å−3]	0.36/−0.55

**Table 2:** Selected bond lengths and angles for complex **4a**.

Atom	Atom	Length/Å		Atom	Atom	Length/Å	
Cu1	Cu1 *	3.0485(5)		Cu2	Cu2 **	3.0501(5)	
Cu1	Cl1	2.1975(5)		Cu2	Cl2	2.1946(5)	
Cu1	O1	1.9435(12)		Cu2	N2	1.9433(15)	
Cu1	O1 *	1.9650(12)		Cu2	O3	1.9304(13)	
Cu1	N1	1.9374(15)		Cu2	O3 **	1.9671(12)	
**Atom**	**Atom**	**Atom**	**Angle/˚**	**Atom**	**Atom**	**Atom**	**Angle/˚**
Cl1	Cu1	Cu1 *	132.188(19)	Cl2	Cu2	Cu2 **	131.859(19)
O1	Cu1	Cu1 *	38.99(4)	N2	Cu2	Cu2 **	127.18(4)
O1 *	Cu1	Cu1 *	38.49(4)	N2	Cu2	Cl2	100.23(5)
O1 *	Cu1	Cl1	99.93(4)	N2	Cu2	O3 **	154.41(6)
O1	Cu1	Cl1	151.53(5)	O3 **	Cu2	Cu2 **	38.07(4)
O1	Cu1	O1 *	77.48(5)	O3	Cu2	Cu2 **	38.93(3)
N1	Cu1	Cu1 *	127.56(5)	O3 **	Cu2	Cl2	100.51(4)
N1	Cu1	Cl1	99.65(5)	O3	Cu2	Cl2	150.29(4)
N1	Cu1	O1 *	155.18(6)	O3	Cu2	N2	92.05(6)
N1	Cu1	O1	92.20(6)	O3	Cu2	O3 **	77.00(5)
Cu1	O1	Cu1 *	102.51(5)	C16	N2	Cu2	116.84(11)
C14	O1	Cu1 *	132.25(11)	C23	N2	Cu2	124.35(13)
C14	O1	Cu1	124.25(11)	Cu2	O3	Cu2 **	103.00(5)
C1	N1	Cu1	118.54(12)	C29	O3	Cu2 **	132.17(11)
C8	N1	Cu1	123.93(13)	C29	O3	Cu2	123.37(11)

Symmetry transformations used to generate equivalent atoms: * 1-x,1-y,-z; ** 2-x,1-y,1-z.

**Table 3:** UV–Vis spectral data of the interaction with ct-DNA, calculated DNA-binding constants (K*_b_*), UV band *λ* (nm) and decrease of intensity (ΔA/A_0_) in %.

Complex	K_b_ [M^−1^]	λ (nm)	(ΔA/A_0_, %)
**4a**	1.43 (±0.84) × 10^5^	369	32
**4b**	1.02 (±0.82) × 10^5^	369	26
**4c**	3.25 (±0.83) × 10^5^	370	28

**Table 4:** EB–DNA fluorescence (%), calculated Stern–Volmer quenching constant (*K_sv_*) and quenching rate constant *k_q_* of complexes **4a**–**c**.

Complex	Δ I/I_0_ [%]	*K_sv_* [M^−1^] × 10^3^	*k_q_* [M^−1^ s^−1^] × 10^11^
**4a**	45.7	4.58 (±0.10)	1.98 (±0.05)
**4b**	40.1	3.61 (±0.09)	1.56 (±0.04)
**4c**	40.7	3.82 (±0.12)	1.66 (±0.05)

**Table 5:** Quantitative evaluation of the nuclease activity of copper(II) complexes.

Complex	c (M)	Form I (%)	Form II (%)	Form III (%)
**4a**	0	90.7	9.7	0
	1 × 10^−3^	58.8	41.2	0
	3 × 10^−3^	53.8	46.2	0
	5 × 10^−3^	0	0	100
**4b**	0	89.7	10.3	0
	1 × 10^−3^	58.5	41.5	0
	3 × 10^−3^	56.2	43.8	0
	5 × 10^−3^	48.2	51.8	0
**4c**	0	93.6	6.4	0
	1 × 10^−3^	49.8	50.2	0
	3 × 10^−3^	0	100	0
	5 × 10^−3^	4.7	95.3	0

**Table 6:** The values of the dynamic quenching constant (*K_sv_*), quenching constant (*k*_q_) association binding constant (*K_BSA_*) and *n* (number of binding sites for albumin) obtained for the interaction of **4a**–**c** with bovine serum albumin.

Complex	K_sv_ (M^−1^) × 10^4^	k_q_ (M^−1^s^−1^) × 10^12^	K_BSA_ (M^−1^) × 10^4^	*n*
**4a**	7.16 (±0.03)	7.16 (±0.03)	6.66 (±0.30)	1.03
**4b**	6.39 (±0.06)	6.39 (±0.06)	5.37 (±0.25)	0.94
**4c**	4.86 (±0.15)	4.86 (±0.15)	4.77 (±0.20)	1.16

**Table 7:** Absorbance values for 570 nm and 600 nm, along with the calculated reduction percentage.

Sample	Absorbance * 570 nm	Absorbance * 600 nm	Reduction Percentage (%)	Cell Death (%)
Positive control C+	1.10	0.12	88.6	12.4
Negative control C−	0.69	0.93	4.4	95.6
**4a** (1 mM)	0.55	0.25	32.9	67.1
**4a** (3 mM)	0.44	0.5	8.4	91.6
**4a** (5 mM)	0.32	0.26	3.5	96.5
**4b** (1 mM)	0.75	0.69	24.0	76.0
**4b** (3 mM)	0.69	0.88	7.4	92.6
**4b** (5 mM)	0.63	0.90	1.0	99.0
**4c** (1 mM)	0.60	0.77	5.2	94.8
**4c** (3 mM)	0.40	0.44	8.5	91.5
**4c** (5 mM)	0.33	0.27	2.6	97.4

* Average of 3 measurements.

**Table 8:** IC_50_ values (μM) against A549 and HepG2 cancer cells and HEL fibroblasts.

Compound	IC_50_—A549	IC_50_—HepG2	IC_50_—HEL
**4a**	66.4 ± 6.2	17.9 ± 2.6	65.9 ± 6.9
**4b**	126.9 ± 8.1	32.8 ± 4.9	44.3 ± 3.6
**4c**	72.0 ± 6.5	21.6 ± 4.9	62.9 ± 6.2
cisplatin	26.2 ± 4.1	16.0 ± 3.4	68.4 ± 0.3

**Table 9:** SOD mimetic activity expressed as % of inhibition of radical transfer to INT at concentrations c_1_ = 0.572 mM or c_1_* = 0.286 mM for **4b** and c_2_ = 0.143 mM and IC_50_ values.

Compound	Activity at c_1_/c_1_* (%)	Activity at c_2_ (%)	SOD-IC_50_ (μM)
**4a**	95.8 ± 2.2	14.3 ± 3.3	242 ± 1
**4b**	40.1 ± 1.9 *	35.8 ± 1.3	1090 ± 56
**4c**	98.9 ± 0.5	23.9 ± 1.3	189 ± 11
cystamine	22.1 ± 1.8	2.0 ± 0.0	1700 ± 100

* c_1_* reduced for complex **4b**.

## Data Availability

The original contributions presented in this study are included in the article/Supplementary Materials. Further inquiries can be directed to the corresponding authors.

## Supplementary Materials

The following supporting information can be downloaded at https://www.mdpi.com/article/10.3390/molecules30173461/s1, the supplementary material file contains additional figures and measured data cited in the article text as, for example, Figure S1-1, Table S2-1 or Equation (S3-1).

## Abbreviations

A549	adenocarcinoma human alveolar basal epithelial cell line
BSA	bovine serum albumin
ct-DNA	calf thymus DNA
DMSO	dimethyl sulfoxide
EB	ethidium bromide
HepG2	human liver epithelial cell line, carcinoma
HEL	lung fibroblast cell line, noncancerous
INT	2-(4-Iodophenyl)-3-(4-nitrophenyl)-5-phenyl-2H-tetrazolium chloride
NADH	nicotinamide adenine dinucleotide
MTT	3-[4,5-dimethylthiazol-2-yl]-2,5 diphenyl tetrazolium bromide
pDNA	plasmid DNA
ROS	reactive oxygen species
SOD	superoxide dismutase
