# Supplementary material for: Synthesis, Structure, DNA/BSA Binding, DNA Cleaving, Cytotoxic and SOD Mimetic Activities of Copper(II) Complexes Derived from Methoxybenzylamine Schiff Base Ligands
Source: Molecules. 2025 Aug 22;30(17):3461. doi: 10.3390/molecules30173461 (PMC12430429; doi:10.3390/molecules30173461)
Supplement: Supplementary file 1 [file molecules-30-03461-s001.zip › molecules-3786959-supplementary.pdf]

Synthesis, structure, DNA/BSA binding, DNA cleaving, cytotoxic and SOD mimetic activities of copper(II) complexes derived from methoxybenzylamine Schiff base ligands

Lucia Lintnerová[1,*], Peter Herich[1,2], Jana Korcová[1], Barbora Svitková[3], Flóra Jozefíková,[1,4] Jindra Valentová[1,*]

SUPPLEMENTARY MATERIAL

S0. Ligand and complexes details

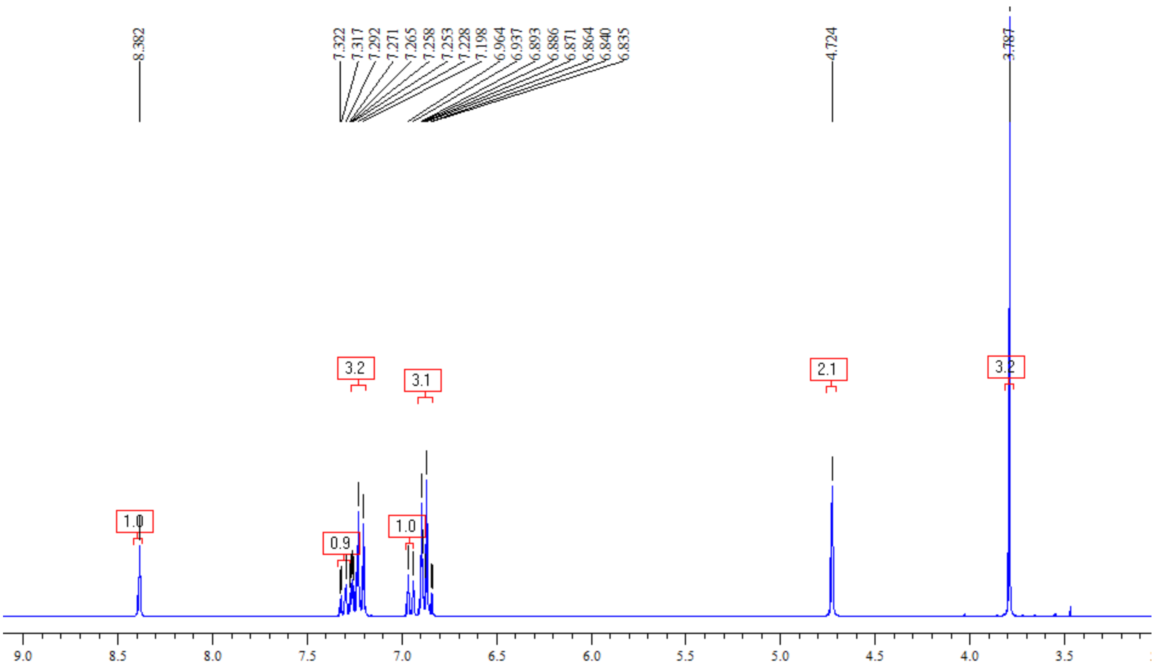

Figure S0-1: ^{1}H NMR spectrum of Schiff base **3c** with hydrogen from CH=N located at 8.382 ppm

Table S0-1: Elemental analysis of prepared complexes

Complex	MW (g/mol)	Calculated %	Measured %
**4a**	339.28	C 53.10; H 4.16; N 4.13	C 53.42; H 4.17; N 4.22
**4b**	544.10	C 66.22; H 5.19; N 5.15	C 66.00; H 5.22; N 5.23
**4c**	339.28	C 53.10; H 4.16; N 4.13	53.44; H 4.19; N 4.19

S0.1. Long-term stability studies

The solubility of the prepared complexes was complete at room temperature in 99+% DMSO up to 4×10^{-3} M and 1×10^{-3} M in 1-5% DMSO aqueous solution.

Stability studies were performed by UV-Vis spectroscopy in the range 200 – 800 nm wavelength in citrate buffer solution. Stability studies were also performed in analogous manner in citrate buffer solution (containing 15 mM of sodium citrate and 150 mM of NaCl at pH = 7.0). The complexes were dissolved in DMSO ($1{\times}10^{-2}$ M) and diluted to $1{\times}10^{-4}$ M with the citrate buffer solution (1% DMSO content). The measurement was done after 24 hours, 48 hours and 72 hours.

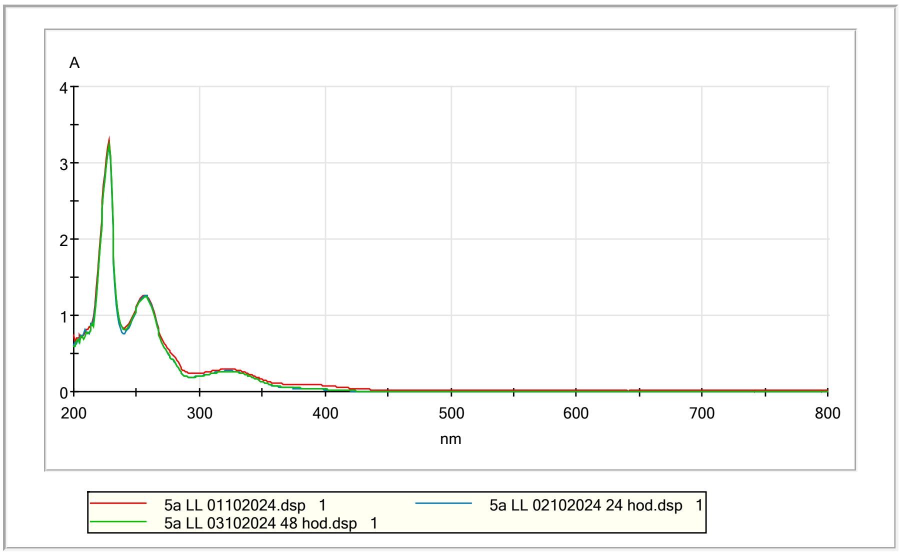

Figure S0-2: UV-Vis spectra of **4a** in citrate buffer solution after 24 hours (red), 48 hours (blue) and 72 hours (green).

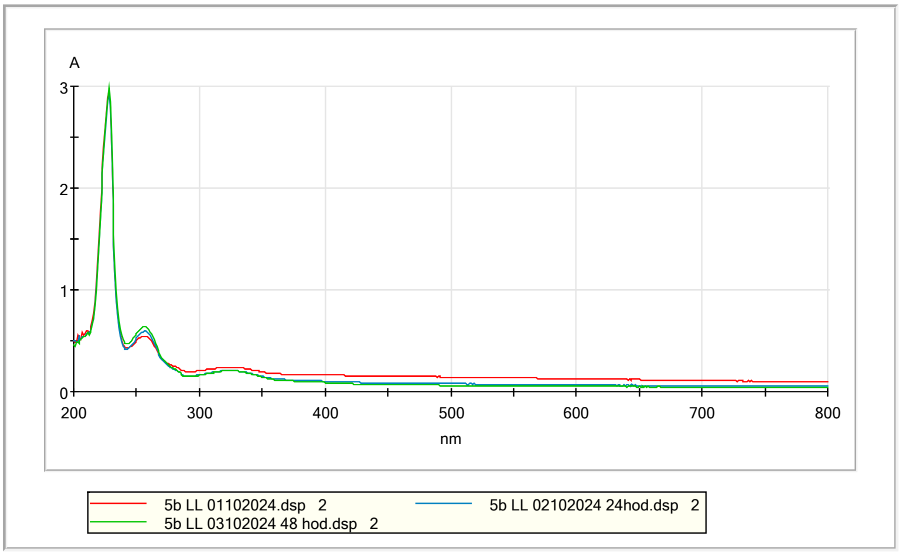

Figure S0-3: UV-Vis spectra of **4b** after 24 hours (red), 48 hours (blue) and 72 hours (green).

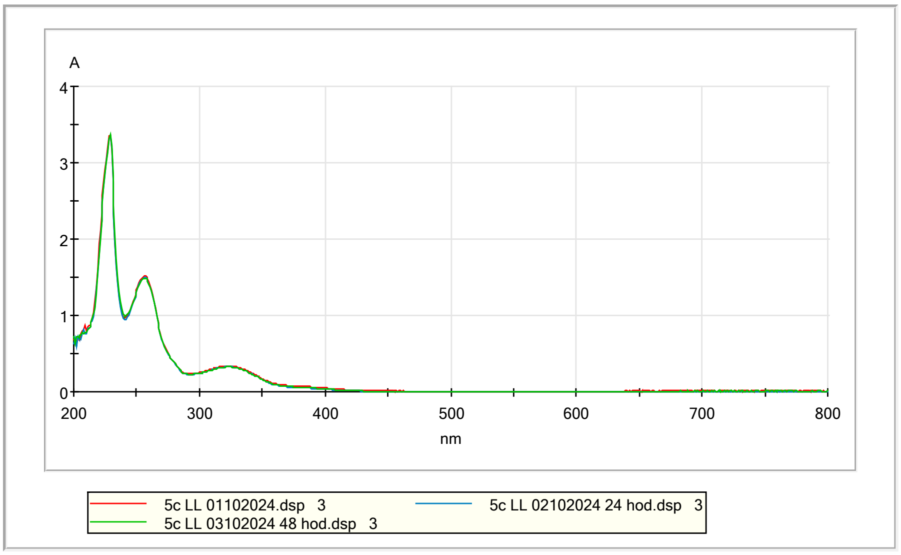

Figure S0-4: UV-Vis spectra of **4c** after 24 hours (red), 48 hours (blue) and 72 hours (green).

Stability studies were performed analogously at 1×10^{-4} M concentration is 99+% DMSO with measurements done after 2 hours (red), 24 hours (blue), 48 hours (green) and 72 hours (purple). A small shift to lower frequency (about 5 cm^{-1}) with no change of peak shape was observed after 48 hours, with no more change afterwards.

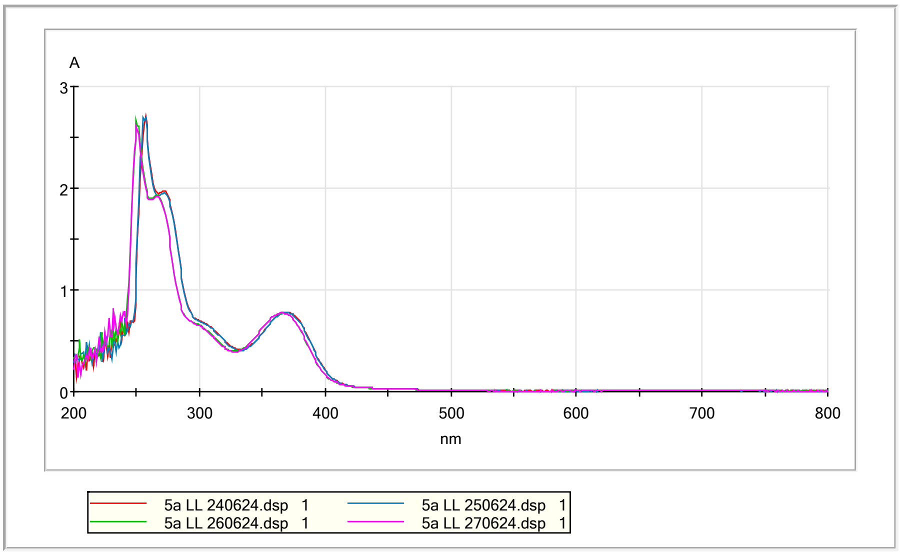

Figure S0-5: UV-Vis spectra of **4a** in DMSO

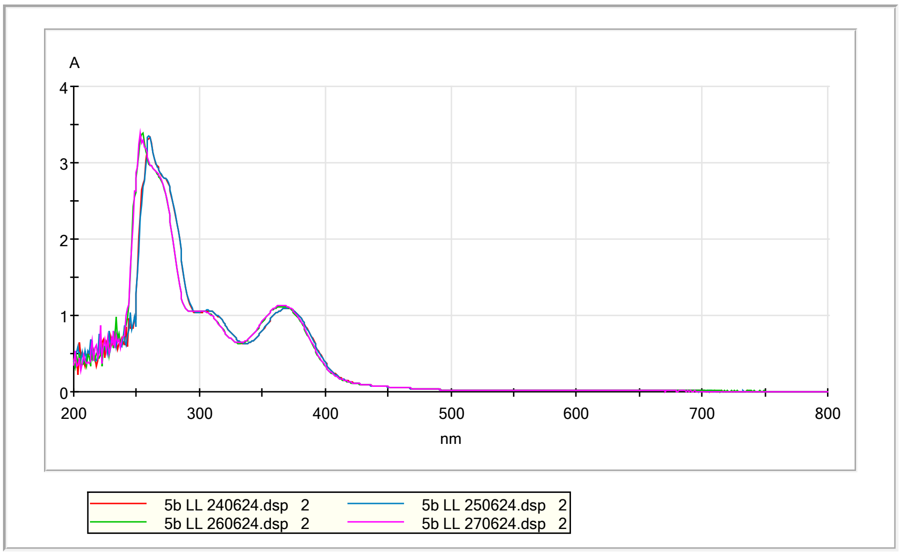

Figure S0-6: UV-Vis spectra of **4b** in DMSO

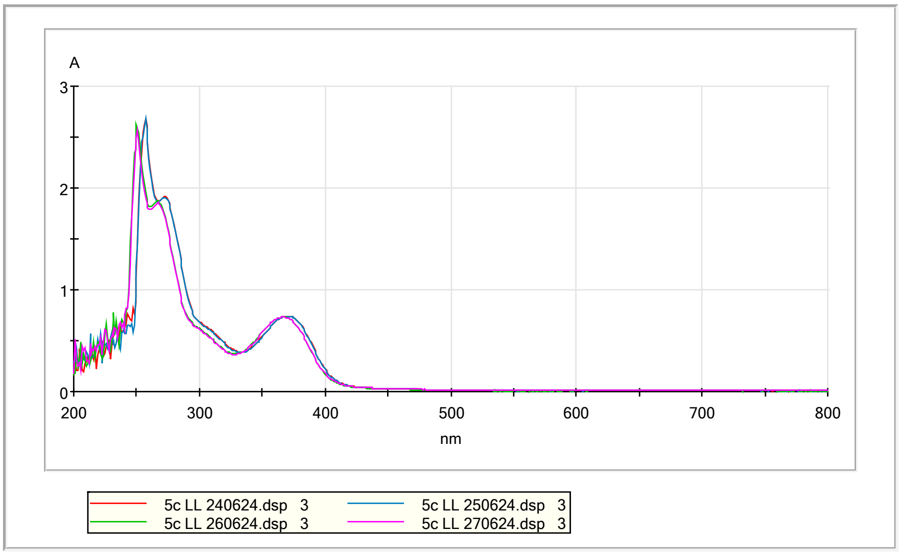

Figure S0-7: UV-Vis spectra of **4c** in DMSO

S0.2. Short-term UV-Vis studies

Comparison of UV-Vis spectrum of ligand and copper salt and complex:

1) a spectrum of separate solutions of the ligand and the copper salt in DMSO each at 1 $\times 10^{-3}$ M (two 250 μl cuvettes each containing one of the components held together in the detection beam pathway)

2) a spectrum of dissolved complex with the same concentration (two cuvettes containing the same) - measured right after the dissolution (happened within max 5 minutes) and after 1 hour.

Comparing the ligand + copper salt combination with the complex spectra, we can see a shift in both spectra below. This suggests that the coordination of ligand to complex is happening, due to differences between green and red spectra. In case of **4c**, a Schiff base ligand complex, also after 1 hour the complex spectra remains the same, confirming the stability of **4c**.

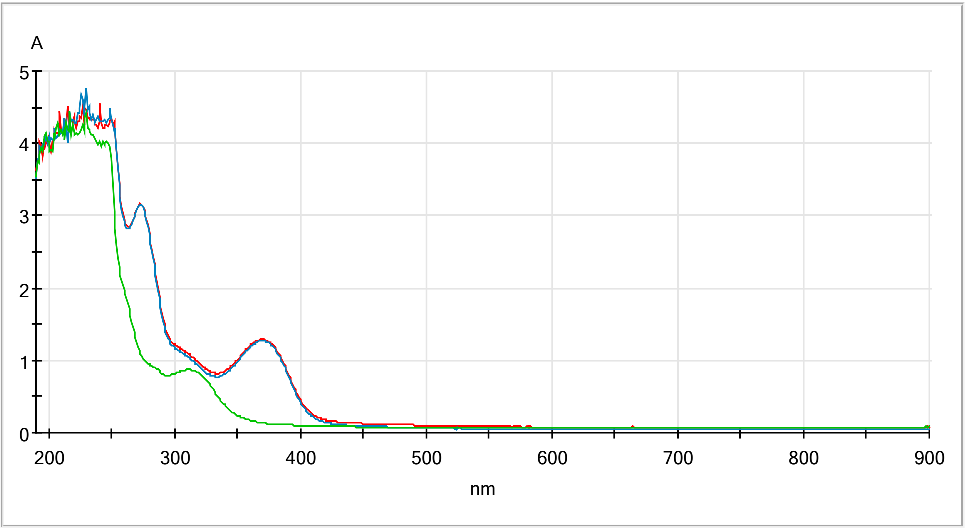

Figure S0-8: UV-Vis spectra of ligand + copper chloride combination (green), complex **4c** right after dissolution (red) and after 1 hour (blue)

S0.3. MS spectra studies of 4b

The elemental analysis of **4b** complex showed the copper : ligand ratio to be 1 : 2, which was different compared to **4a** and **4c**. The structure of 4a was determined by using X-ray crystallography, but since we could not obtain a well diffracting crystal for **4b**, we used HRMS study of this complex to determine the molecular structure. We observed that the complex is present in more forms, including monomer, dimer and aggregates with more than one copper center. There seems to be some equilibrium between a 1:1 form, 2 : 1 form and higher forms at 1 mM.

All spectra were measured in positive mode.

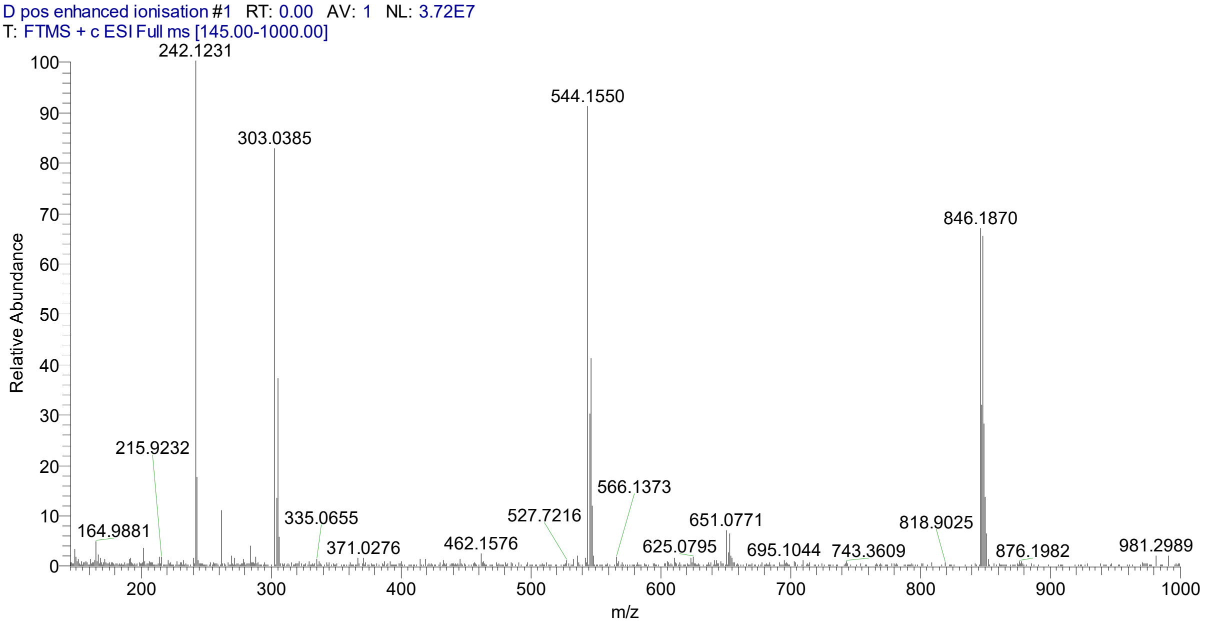

Figure S0-9: MS fragmentation spectrum from for complex **4b**

Fragment 242: ligand+H^+

Fragment 303: ligand+Cu+H^+

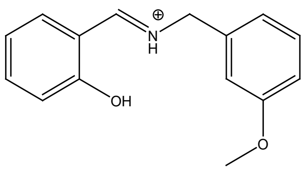

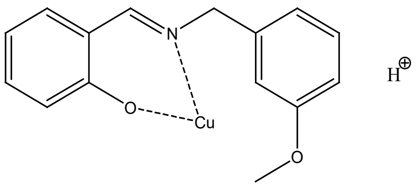

Fragment 544: dimer with 2:1 ratio (ligand : Cu) + H^+

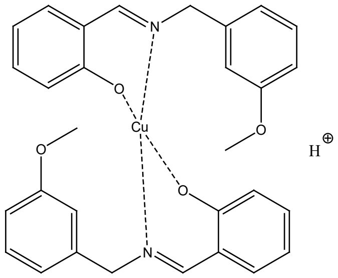

Fragment 846: aggregation of of 3 molecules of ligand and 2 copper centers + H^+

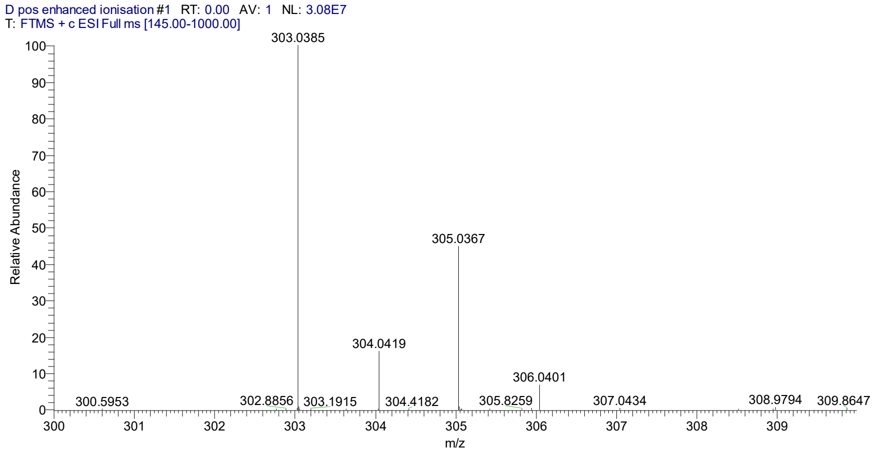

Figure S0-10: Zoom on the surroundings of 303 fragment: most prominent feature is the ration of 303 and 305 fragments in about 3 : 1 ration which would confirm Cu presence due to its isotope Cu-63 and Cu-65 having natural occurring ratio.

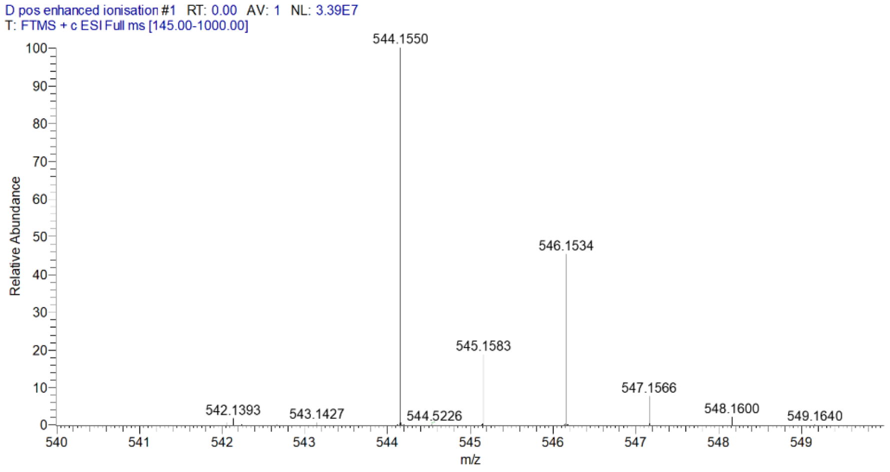

Figure S0-11: Zoom in on the surroundings of 544 fragment. Once again the isotope ratios of copper can be seen.

From the observed, we can assume that the complex **4b** is present in monomer form but also forms aggregates at 1 mM concentration.

S0.4. EPR studies of complexes

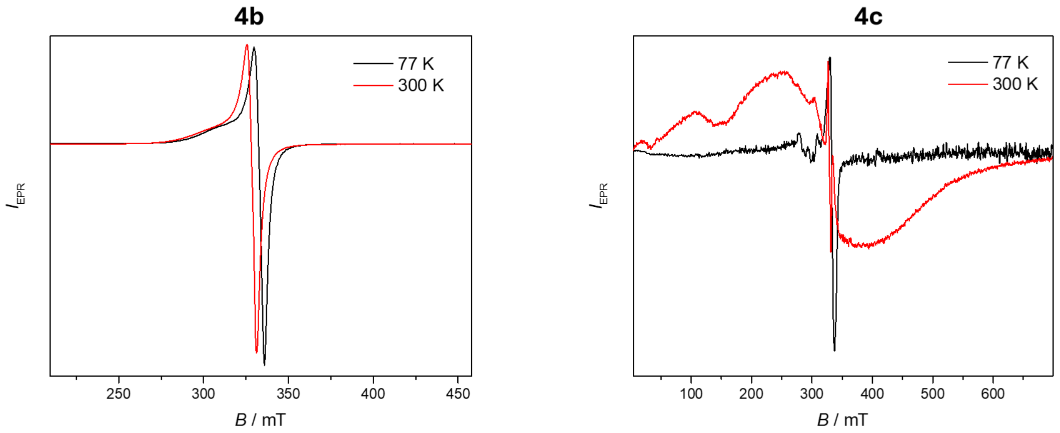

Figure S0-12: Detail of EPR spectrum of solid powder of **4b-c** at 77 K (black line) and at 300 K (red line).

The observed axial copper spectrum of **4a** can be simply simulated using the following spin system parameters g_{II} = 2.279 , $g_{\perp}$ =2.054 , $A_{Cu\ II}$=437 MH:

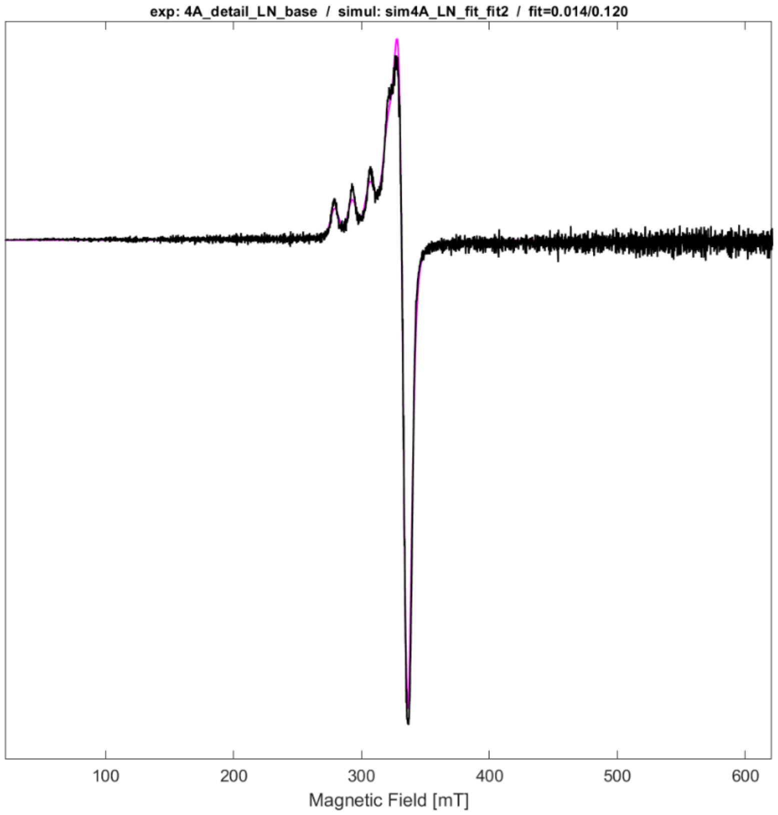

Figure S0-13: Detailed view on EPR spectrum of crystalline powder of **4a** at 77 K (black line) and its simulation (magenta line).

The observed axial copper spectrum can be simply simulated using the following spin system parameters g_{II} = 2.269 , $g_{\perp}$ =2.052 , $A_{Cu\ II}$=482 MHz:

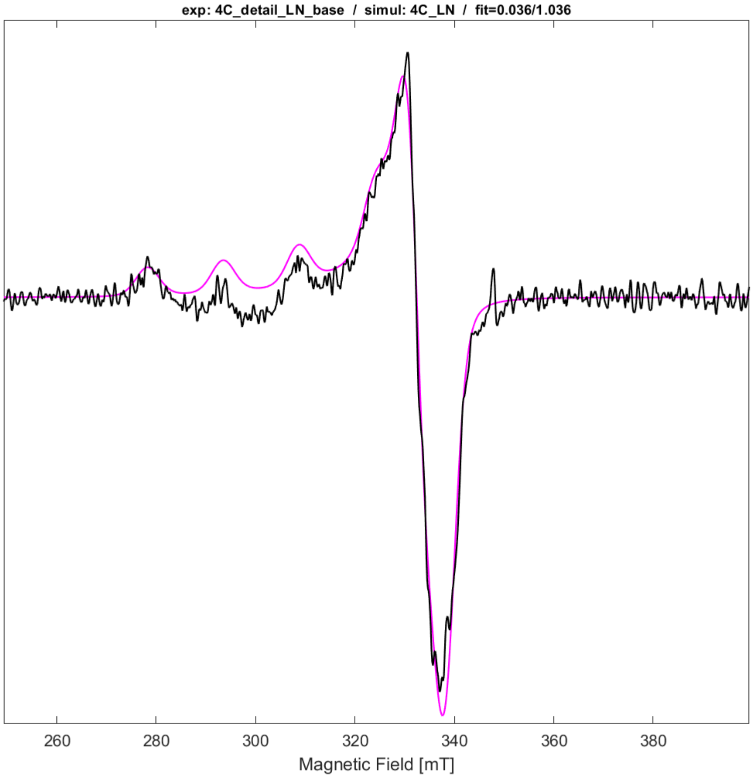

Figure S0-14: Detailed view on solid state EPR spectrum of **4c** at 77 K (black line) and its simulation (magenta line).

S1. X-Ray Structure Analysis

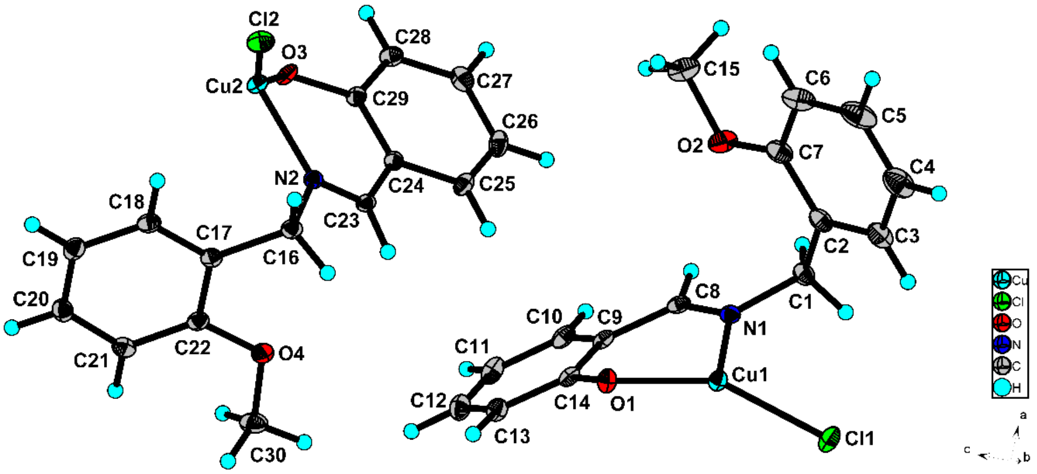

Figure S1-1: The molecular structure (asymmetric parts) of complex **4a** showing the atom-labelling scheme without hydrogen atoms labels for clarity. Displacement ellipsoids are drawn at the 50% probability level.

The crystal structure of complex 4a (Figure S1-2) is stabilized by a system of intermolecular hydrogen bonds and interactions (Figure S1-3).

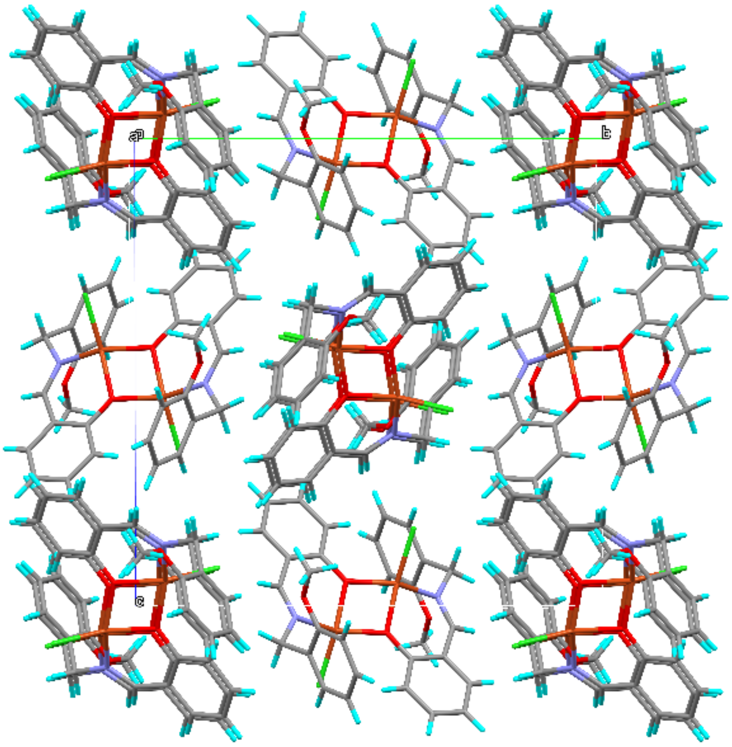

Figure S1-2: The crystal structure of complex **4a** viewed along the **a** axis.

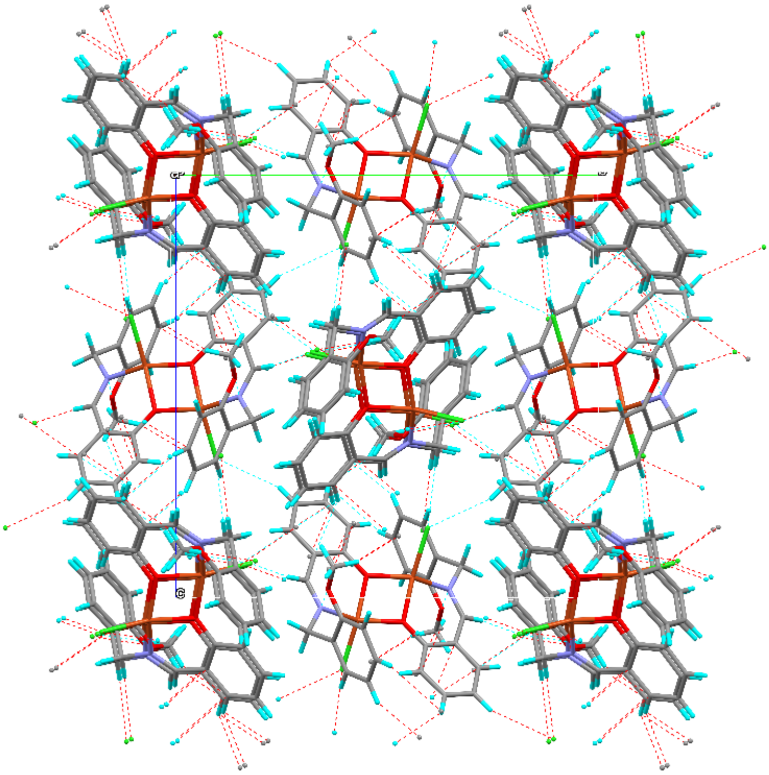

Figure S1-3: The crystal structure of complex **4a** with system hydrogen bond and interactions viewed along the **a** axis.

Table S1-1. Hydrogen bonds and interactions for complex **4a**.

D	H	A	d(D-H)/Å	d(H-A)/Å	d(D-A)/Å	D-H-A/°
C1	H1B	Cl1	0.99	2.84	3.369(2)	114.1
C13	H13	Cl1(i)	0.95	2.86	3.4070(19)	117.6
C16	H16A	Cl2	0.99	2.82	3.3918(19)	117.4
C16	H16B	Cl1(i)	0.99	2.72	3.6578(18)	158.2
C30	H30C	Cl2(ii)	0.98	2.94	3.907(2)	171.5

The cif files were uploaded to CCDC (Cambridge Crystallographic Database Center) under these deposition number: **2367651**

Summary of Data - Deposition Number **2367651**

Compound Name:

Data Block Name: data_ll01

Unit Cell Parameters: a 9.21980(10) b 17.4213(3) c 17.4576(2) P21/c

S2. DNA Cleavage Assay

In the following tables, the percentage (%) expression of the amount of pDNA that has passed through the effect of the nuclease activity of the copper complex from the uncleaved supercoiled form I to the cleaved form II (open circular pDNA) or form III (linear pDNA). Quantitative evaluation was performed using the "gelQUANT software" (Cleaver Scientific, UK). $V_{(tested\ sample)}$ = 20 ul ; $c_{(pDNA)}$ = 250 ng

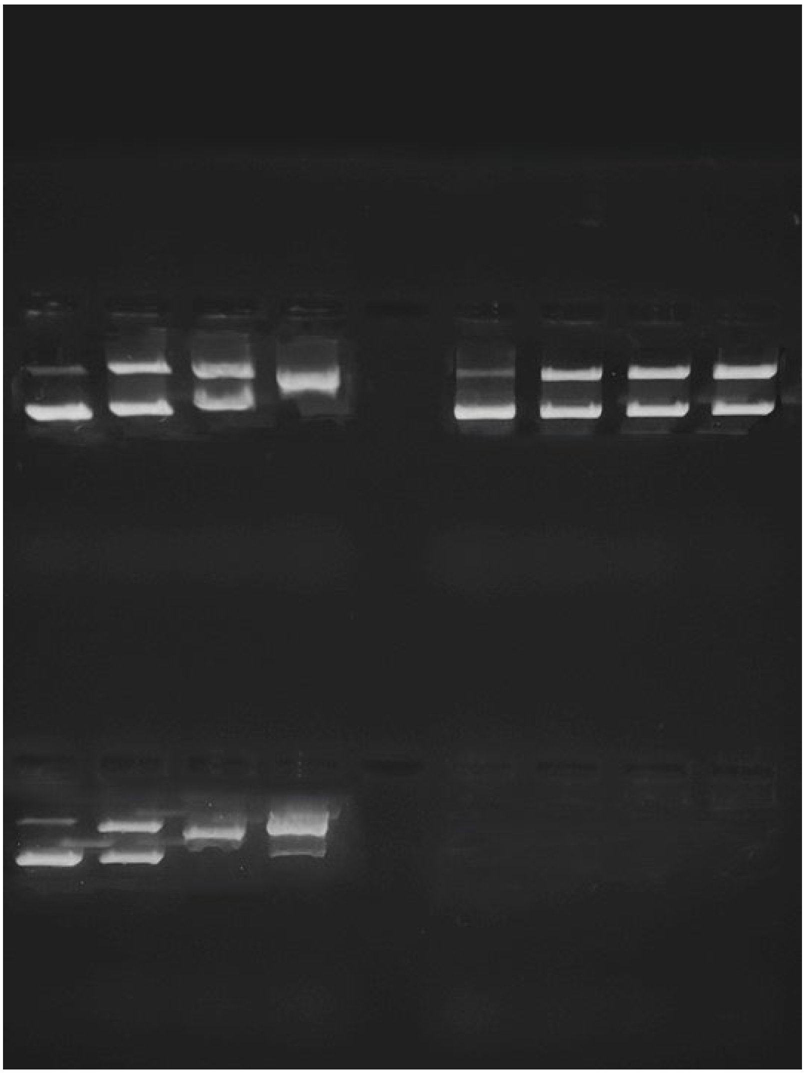

Figure S2-1: The original gel scan: upper left lines 1 – 4 complex **4a** (0 mM, 1 mM, 3mM, 5mM), upper lines 6 – 9 complex **4b** (0 mM, 1 mM, 3mM, 5mM), lower lines 1 – 4 complex **4c** (0 mM, 1 mM, 3mM, 5mM).

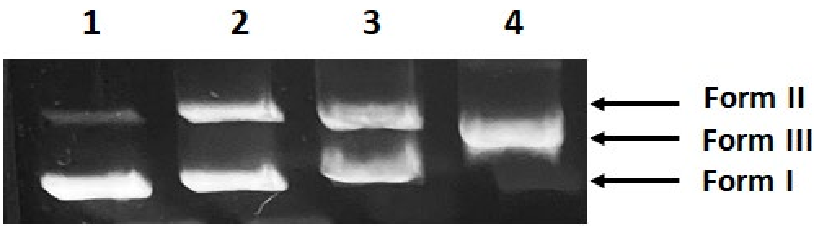

ELFO line	Complex 4a	Incubation time - 3h		
		Form I. (%)	Form II. (%)	Form III. (%)
**1**	**c (mM)**	90,7 (226,75 ng)	9,7 (24,25 ng)	0
**2**	**1**	58,8 (147 ng)	41,2 (103 ng)	0
**3**	**3**	53,8 (134,5 ng)	46,2 (115,5 ng)	0
**4**	**5**	0	0	100 (250 ng)

Table S2-1: Quantitative evaluation of nuclease activity of the copper complex **4a**.

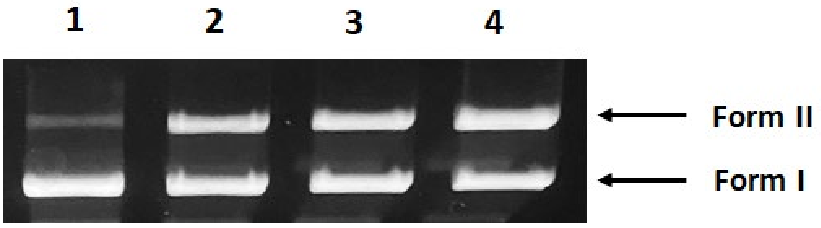

ELFO line	Complex 4b	Incubation time - 3h		
		Form I. (%)	Form II. (%)	Form III. (%)
**1**	**c (mM)**	89,7 (224,25 ng)	10,3 (25,75 ng)	0
**2**	**1**	58,5 (146,25 ng)	41,5 (103,75 ng)	0
**3**	**3**	56,2 (140,5 ng)	43,8 (109,5 ng)	0
**4**	**5**	48,2 (120,5 ng)	51,8 (129,5 ng)	0

Table S2-2: Quantitative evaluation of nuclease activity of the copper complex**4b**.

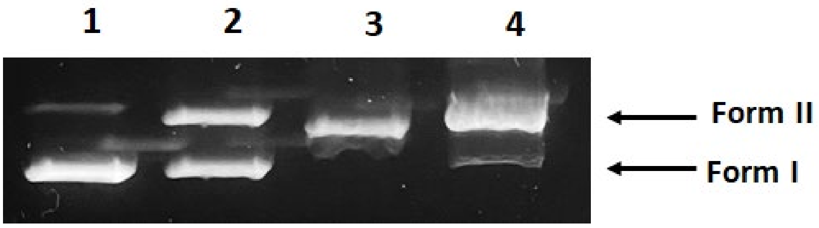

ELFO line	Complex 4c	Incubation time - 3h		
		**Form I. (%)**	**Form II. (%)**	**Form III. (%)**
**1**	**c (mM)**	93,6 (234 ng)	6,4 (16 ng)	0
**2**	**1**	49,8 (124,5 ng)	50,2 (125,5 ng)	0
**3**	**3**	0	100 (250 ng)	0
**4**	**5**	4,7 (11,75 ng)	95,3 (238,25 ng)	0

Table S2-3: Quantitative evaluation of nuclease activity of the copper complex **4c**.

S3. DNA Binding Assays

DNA-binding study by absorption titration

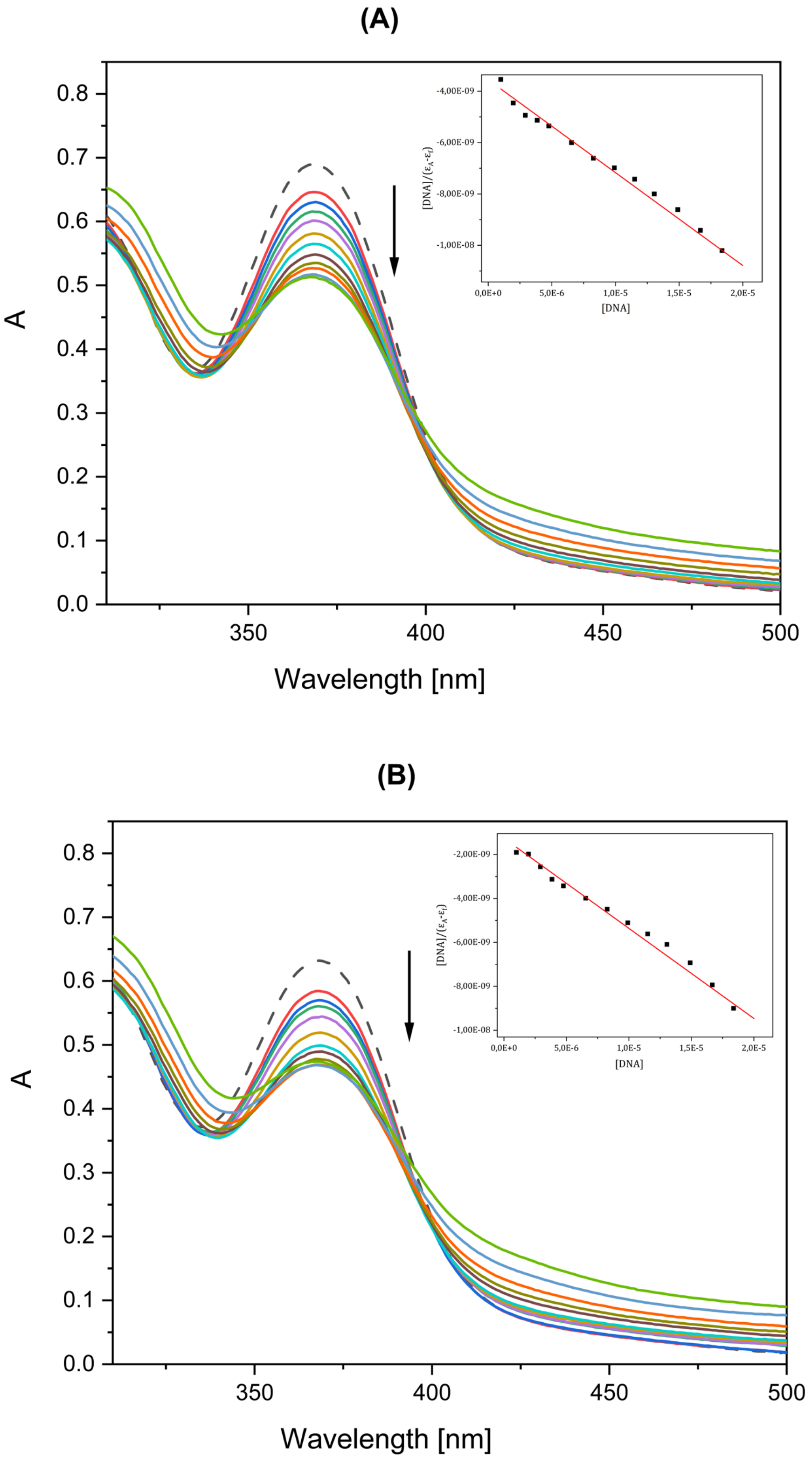

Figure S3-1: UV-Vis spectra of DMSO solution of complex **4b (A)** and **4c (B)** in the absence (dashed line) and presence (solid line) of increasing amounts of ct-DNA (*r* = [DNA]/[complex] = 0 - 2.1). The arrows

show changes in intensity upon the addition of increasing amounts of ct-DNA. The insert showed the least-squares fit of [DNA]/ε_A-ε_f vs [DNA] for complex according to the Wolfe-Shimer equation.

Competitive studies with EB-DNA

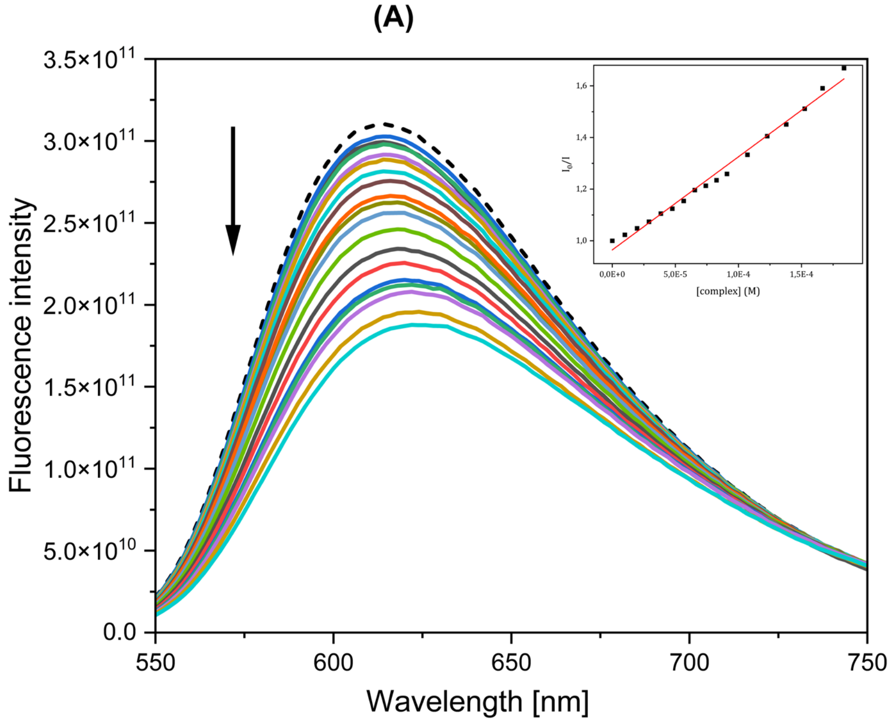

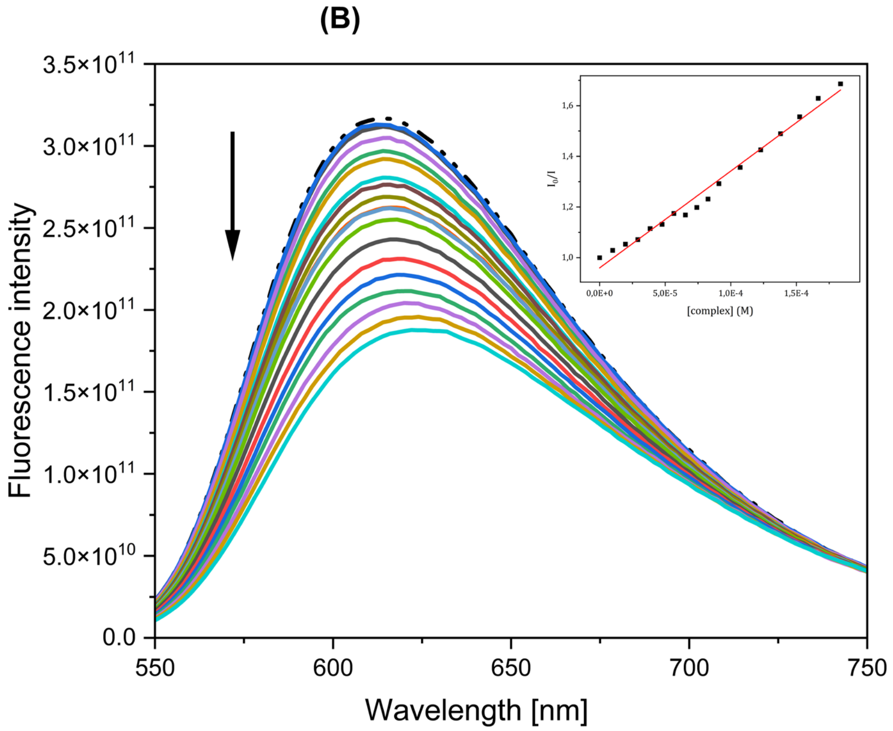

Figure S3-2 Emission fluorescence spectra of EB-DNA in the buffer solution in the presence of increasing amount of complex **4b (A)** and **4c (B)**. The concentration of added complexes ranged from 0

to 0.16 mM (concentration increment 1.6 × 10⁻² mM, indicated by different color of the curve). The arrow indicated the changes in fluorescence at increasing amounts of the complex. Insert graph shows the plot of I_0/I vs [complex].

S4. Albumin binding studies

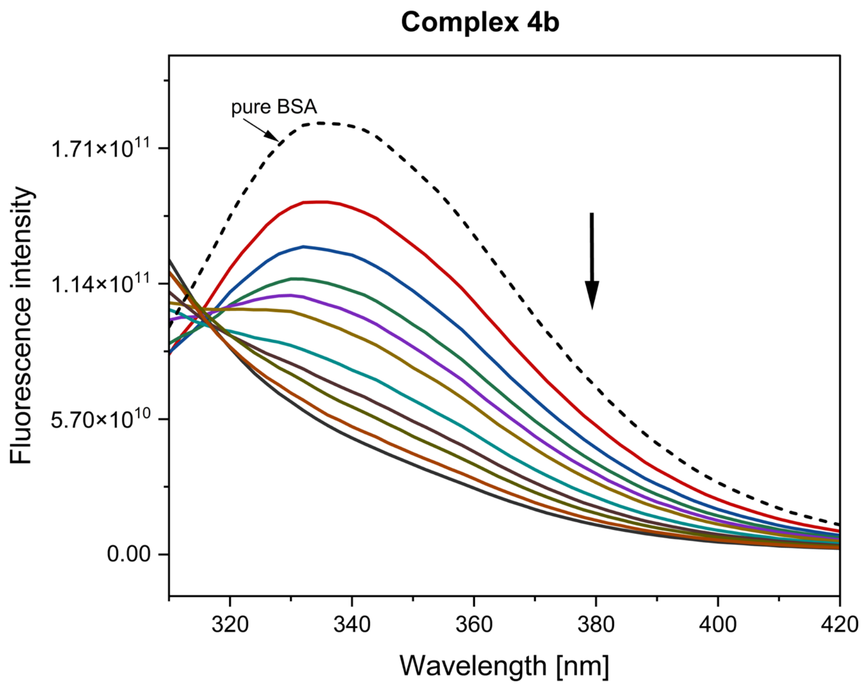

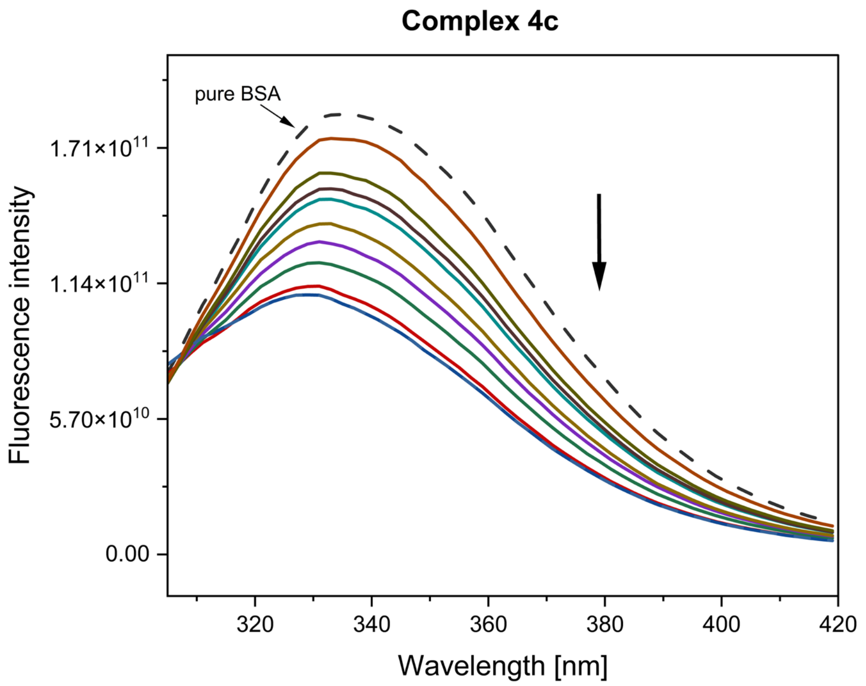

Figure S4-1: Fluorescence emission spectra of BSA in buffer solution in the presence of increasing concentrations of the complexes **4b** and **4c**. The arrow indicates the changes in fluorescence with

increasing amounts of the complex. The concentration of added complexes ranged from 0 to 1.6 × 10^{-2} mM (concentration increment 1.6 μM, indicated by different color of the curve). The dash line showed the intensity in the absence of complex.

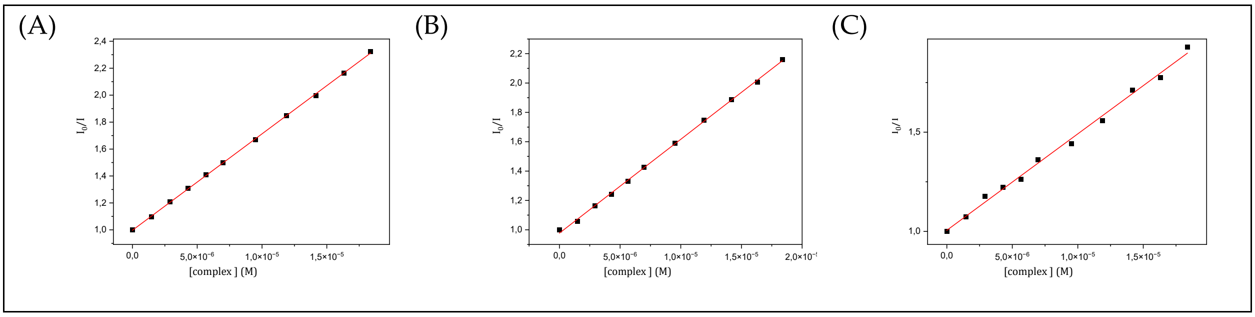

Figure. S4-2. Stern-Volmer quenching plot of BSA for complex (A) **4a**, (B) **4b**, (C) **4c**.

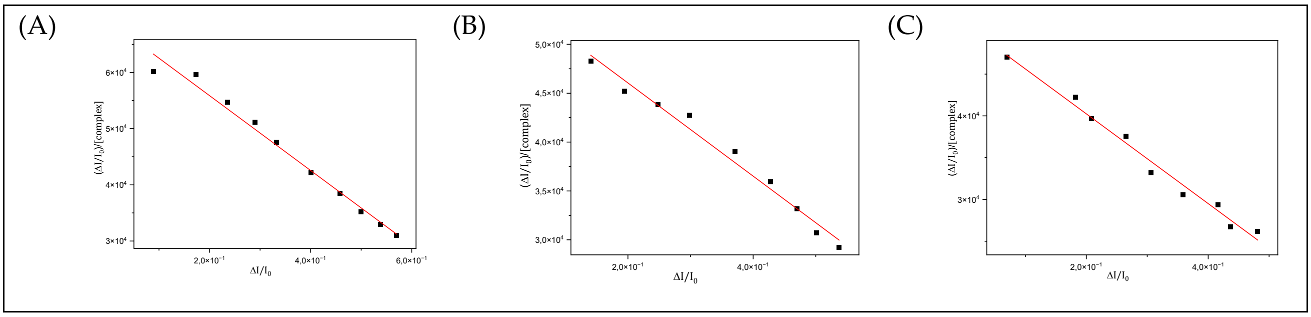

Figure. S4-3. Scatchard plot of BSA for complex (A) **4a**, (B) **4b**, (C) **4c**.

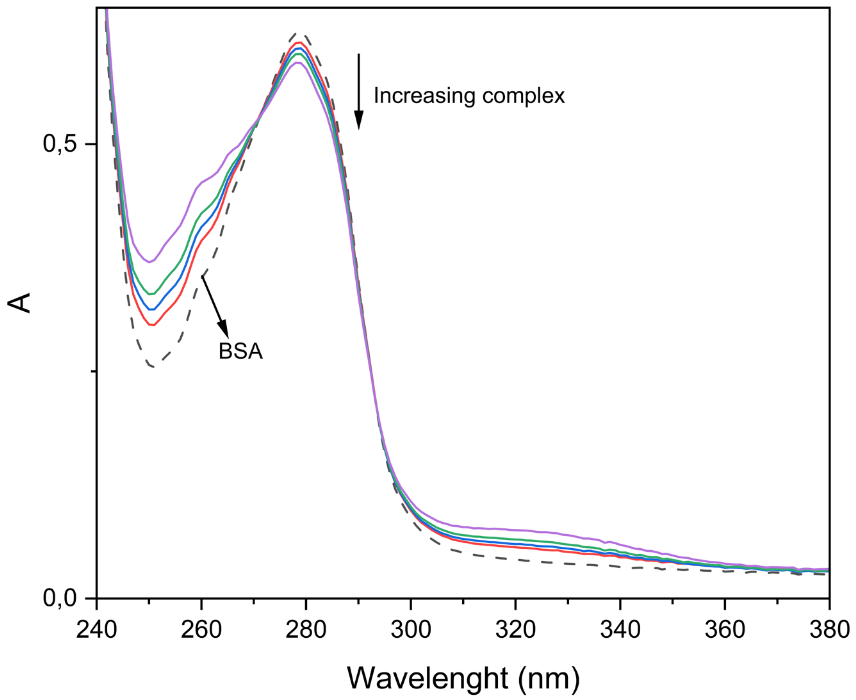

Figure S4-4: Representative UV-VIS absorption spectra of BSA in the absence or presence of the complex **4c**; [BSA] = 3 μM and [complex **4c**] = 0 – 12.5 μM.

S5. Resazurin Cytotoxicity Assay

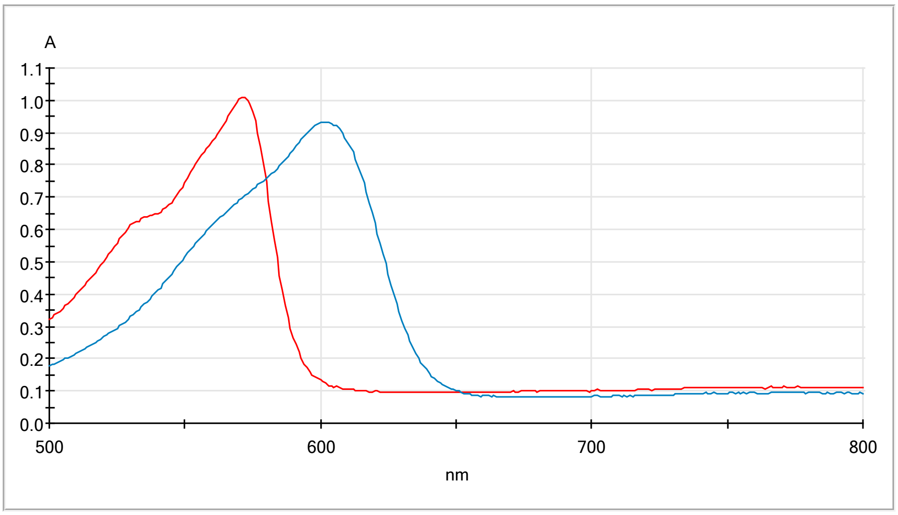

Figure S5-1: Determination of the ability of *S. cerevisiae* live cells to reduce Resazurin$_{ox}$ into Resorufin. Control samples. UV-VIS measurement range λ = 500 – 800 nm.

Blue: C- Negative Control, λ max = 601 nm (*S. cerevisiae* 1x10^9 cells/ml + 1 ml 0.9% NaCl + 0.1 ml Resazurin$_{OX}$) measured in time t_0

Red: C+ Positive Control, λ max = 572 nm (*S. cerevisiae* 1x10^9 cells/ml + 1 ml 0.9% NaCl + 0.1 ml Resazurin$_{OX}$ – which was metabolized in the mitochondria of healthy cells to Resazurin$_{RED}$ - Resorufin) measured in time t =3h

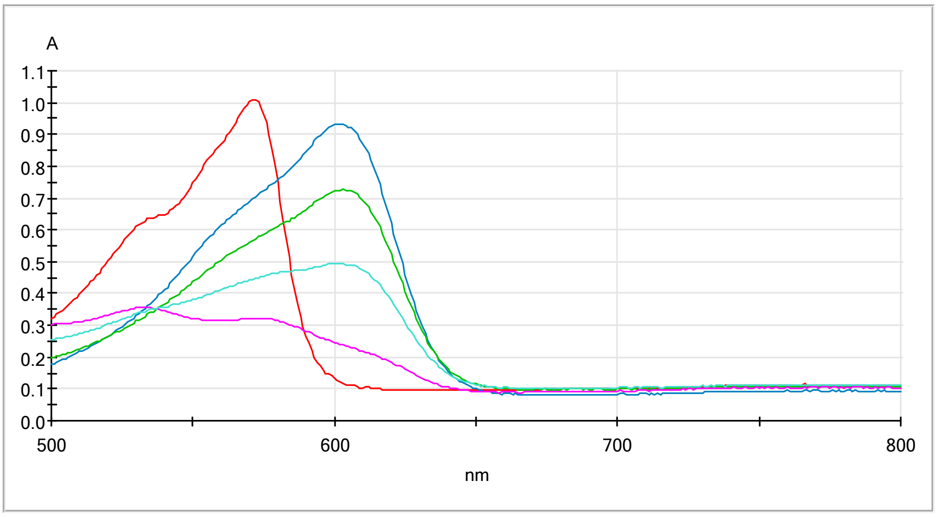

Figure S5-2: Determination of the ability of *S. cerevisiae* live cells to reduce Resazurin$_{ox}$ into Resorufin after 3h incubation with copper complex **4a**.

Blue: C- Negative Control, λ max = 601 nm (*S. cerevisiae* 1x10^9 cells/ml + 1 ml 0.9% NaCl + 0.1 ml Resazurin$_{OX}$) measured in time t_0

Red: C+ Positive Control, λ max = 572 nm (*S. cerevisiae* 1x10^9 cells/ml + 1 ml 0.9% NaCl + 0.1 ml Resazurin$_{OX}$) measured in time t =3h

Green: **5a** 1 mM (*S. cerevisiae* 1x10^9 cells /ml + 0.9 ml 0.9% NaCl + 0.1 ml **5a** + 0.1 ml Resazurin$_{OX}$) measured in time t =3h

Cyan: **5a** 3 mM (*S. cerevisiae* 1x10^9 cells /ml + 0.7 ml 0.9% NaCl + 0.3 ml **5a** + 0.1 ml Resazurin$_{OX}$) measured in time t =3h

Magenta: **5a** 5 mM (*S. cerevisiae* 1x10^9 cells /ml + 0.5 ml 0.9% NaCl + 0.5 ml **5a** + 0.1 ml Resazurin$_{OX}$) measured in time t =3h

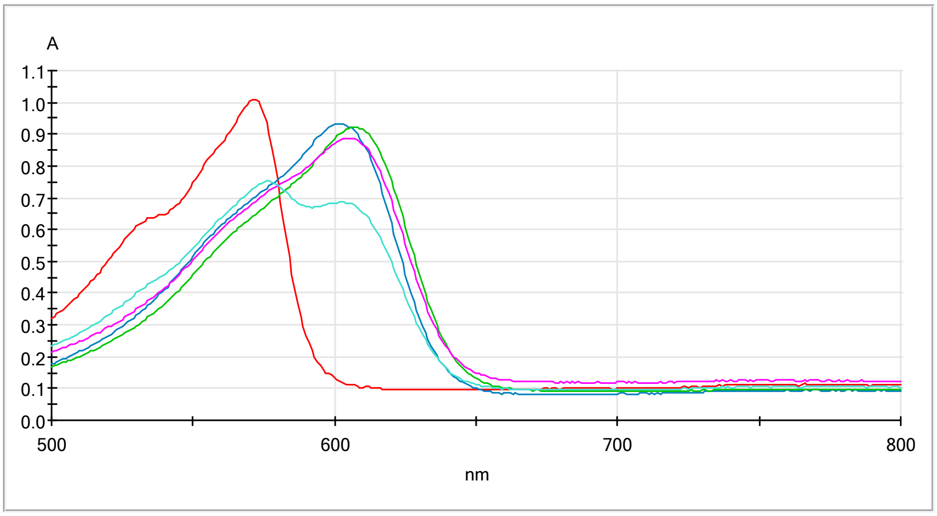

Figure S5-3: Determination of the ability of *S. cerevisiae* live cells to reduce Resazurin$_{ox}$ into Resorufin after 3h incubation with copper complex **4b**.

Blue: C- Negative Control, λ max = 601 nm (*S. cerevisiae* 1x10^9 cells/ml + 1 ml 0.9% NaCl + 0.1 ml Resazurin$_{OX}$) measured in time t_0

Red: C+ Positive Control, λ max = 572 nm (*S. cerevisiae* 1x10^9 cells/ml + 1 ml 0.9% NaCl + 0.1 ml Resazurin$_{OX}$) measured in time t =3h

Cyan: **5b** 1 mM (*S. cerevisiae* 1x10^9 cells /ml + 0.9 ml 0.9% NaCl + 0.1 ml **5b** + 0.1 ml Resazurin$_{OX}$) measured in time t =3h

Magenta: **5b** 3 mM (*S. cerevisiae* 1x10^9 cells /ml + 0.7 ml 0.9% NaCl + 0.3 ml **5b** + 0.1 ml Resazurin$_{OX}$) measured in time t =3h

Green: **5b** 5 mM (*S. cerevisiae* 1x10^9 cells /ml + 0.5 ml 0.9% NaCl + 0.5 ml **5b** + 0.1 ml Resazurin$_{OX}$) measured in time t =3h

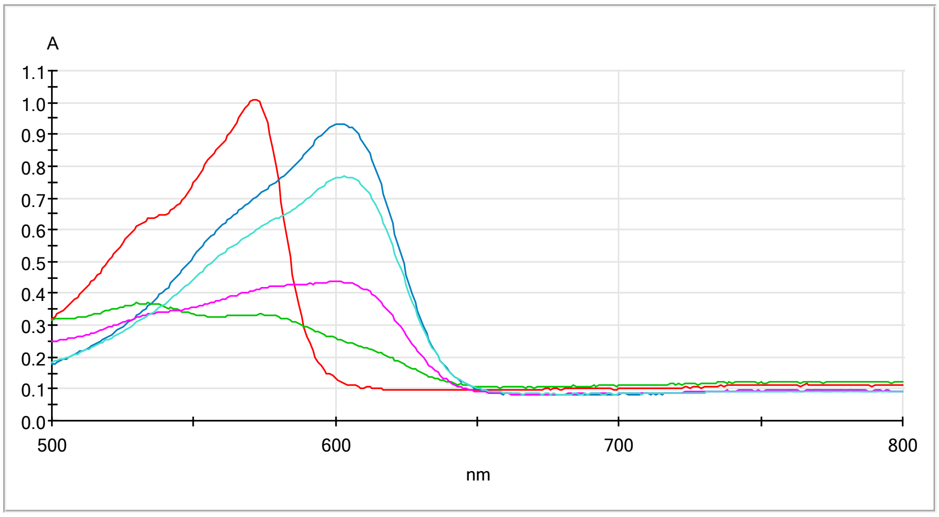

Figure S5-4: Determination of the ability of *S. cerevisiae* live cells to reduce Resazurin$_{ox}$ into Resorufin after 3h incubation with copper complex **4c**.

Blue: C- Negative Control, λ max = 601 nm (*S. cerevisiae* 1x10^9 cells/ml + 1 ml 0.9% NaCl + 0.1 ml Resazurin$_{OX}$) measured in time t_0

Red: C+ Positive Control, λ max = 572 nm (*S. cerevisiae* 1x10^9 cells/ml + 1 ml 0.9% NaCl + 0.1 ml Resazurin$_{OX}$) measured in time t =3h

Cyan: **5c** 1 mM (*S. cerevisiae* 1x10^9 cells /ml + 0.9 ml 0.9% NaCl + 0.1 ml **5c** + 0.1 ml Resazurin$_{OX}$) measured in time t =3h
Magenta: **5c** 3 mM (*S. cerevisiae* 1x10^9 cells /ml + 0.7 ml 0.9% NaCl + 0.3 ml **5c** + 0.1 ml Resazurin$_{OX}$) measured in time t =3h
Green: **5c** 5 mM (*S. cerevisiae* 1x10^9 cells /ml + 0.5 ml 0.9% NaCl + 0.5 ml **5c** + 0.1 ml Resazurin$_{OX}$) measured in time t =3h

S6. SOD Mimetic Activity Assay

For each measurement, an initial solution of the sample of a specific concentration was prepared (4×10^{-3} – 1×10^{-4} M) in DMSO, also initial solutions of INT (4 M) in tetraborate buffer (pH = 7.1) and a saturated solution of KO_2 in DMSO. From these a set of diluted solutions of volume 3.5 ml was made:

A: blank solution containing only INT dye (0.5 ml of INT solution and 3 ml of DMSO),

B: 3-5 control solutions containing INT dye and KO_2 (0.5 ml of INT solution, 0.5 ml of KO_2 solution and 2.5 ml of DMSO),

C: 3 sample solutions containing all three compounds - the complex, INT dye and KO_2 (0.5 ml of INT solution, 0.5 ml of initial complex solution of concentration 4 – 0.1 mM, 0.5 ml of KO_2 solution and 2 ml of DMSO)

D: sample control solution containing the complex and KO_2 (0.5 ml of complex solution of concentration 4 – 0.1 mM, 0.5 ml of KO_2 solution and 2.5 ml of DMSO).

In sample solutions and sample control solutions (C and D) the concentration of the sample was diluted 7 times (0.5 ml of initial sample solution in 3.5 ml of overall mixture). 200 μl of the solutions were moved into a micro-titration plate and the absorbance at 500 nm was measured in parallel in all four types of solutions. The resulting inhibition percent was calculated based on the formula: 100 – [(absorbance of C – absorbance of D) / average absorbance of B] x 100. The result of each measurement was the average value from the 3 sample solutions with a standard deviation.

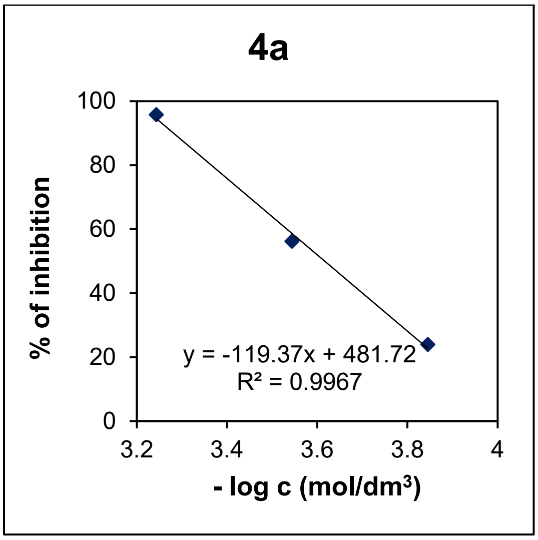

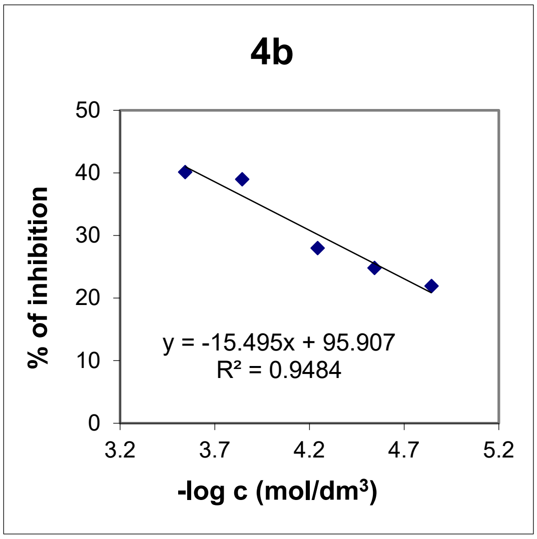

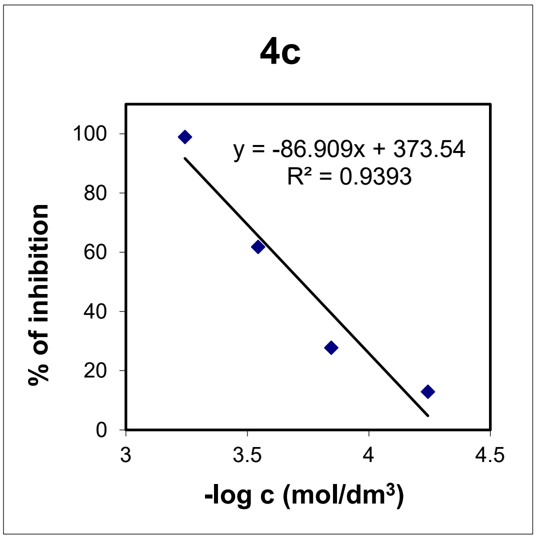

Figure S6-1: SOD-IC50 determination of **4a-c**
